# Bursting Dynamics Based on the Persistent Na^+^ and Na^+^/K^+^ Pump Currents: A Dynamic Clamp Approach

**DOI:** 10.1523/ENEURO.0331-22.2023

**Published:** 2023-08-11

**Authors:** Ricardo Erazo-Toscano, Mykhailo Fomenko, Samuel Core, Ronald L. Calabrese, Gennady Cymbalyuk

**Affiliations:** 1 Neuroscience Institute, Georgia State University, Atlanta, 30302 GA; 2 Department of Biology, Emory University, Atlanta, 30322 GA

**Keywords:** central pattern generator, interneuron, invertebrate, oscillatory networks, electrophysiology, bursting neuron, intracellular Na^+^ concentration

## Abstract

Life-supporting rhythmic motor functions like heart-beating in invertebrates and breathing in vertebrates require an indefatigable generation of a robust rhythm by specialized oscillatory circuits, central pattern generators (CPGs). These CPGs should be sufficiently flexible to adjust to environmental changes and behavioral goals. Continuous self-sustained operation of bursting neurons requires intracellular Na^+^ concentration to remain in a functional range and to have checks and balances of the Na^+^ fluxes met on a cycle-to-cycle basis during bursting. We hypothesize that at a high excitability state, the interaction of the Na^+^/K^+^ pump current, I_pump_, and persistent Na^+^ current, I_NaP_, produces a mechanism supporting functional bursting. I_NaP_ is a low voltage-activated inward current that initiates and supports the bursting phase. This current does not inactivate and is a significant source of Na^+^ influx. I_pump_ is an outward current activated by [Na^+^]_i_ and is the major source of Na^+^ efflux. Both currents are active and counteract each other between and during bursts. We apply a combination of electrophysiology, computational modeling, and dynamic clamp to investigate the role of I_pump_ and I_NaP_ in the leech heartbeat CPG interneurons (HN neurons). Applying dynamic clamp to introduce additional I_pump_ and I_NaP_ into the dynamics of living synaptically isolated HN neurons in real time, we show that their joint increase produces transition into a new bursting regime characterized by higher spike frequency and larger amplitude of the membrane potential oscillations. Further increase of I_pump_ speeds up this rhythm by shortening burst duration (BD) and interburst interval (IBI).

## Significance Statement

Central pattern generators (CPGs) are neuronal networks that control rhythmic motor functions such as breathing and walking. Synaptic and membrane properties enable participating neurons to generate functional bursting patterns; intracellular Na^+^ concentration reflects the intensity of the spiking activity during bursts and regulates the Na^+^/K^+^ pump current playing a critical role in sculpting these patterns by providing negative feedback in response to excitation. Here, we show that the dynamic interaction of Na^+^/K^+^ pump current with persistent Na^+^ current offers a mechanism for the generation of a robust and flexible pattern of bursting activity. We provide physiological data and present a simple model that explains the underlying dynamics of the oscillatory mechanism.

## Introduction

Life-sustaining rhythmic motor behaviors such as breathing are controlled by oscillatory neuronal networks known as central pattern generators (CPGs). The oscillatory patterns in CPGs arise from the interaction of endogenous membrane and synaptic currents (Harris-Warrick, 1993; Marder and Calabrese, 1996; Prinz et al., 2004b). CPGs must be flexible and adapt to behavioral goals and environmental conditions by changing the motor output period and spike frequency (Harris-Warrick and Marder, 1991; Katz, 1996). Many studies have demonstrated that membrane and synaptic properties are subject to neuromodulation that implements changes in the rhythmic patterns. Among these properties, neuromodulators such as myomodulin and dopamine target the activity of the Na^+^/K^+^ pump (Stimers and Dobretsov, 1998; Dobretsov and Stimers, 2005; Tobin and Calabrese, 2005; Wang et al., 2012; Kueh et al., 2016; Picton et al., 2017b). The Na^+^/K^+^ pump sculpts the activity of CPG neurons by generating an outward current reflecting Na^+^ entry associated with spiking activity. This feature, taken together with its voltage independence, distinguishes the pump current from typical voltage-gated membrane currents (Dobretsov and Stimers, 2005; Kueh et al., 2016; Picton et al., 2017b; Ellingson et al., 2021; Sharples et al., 2021). The biophysical mechanisms underlying the contribution of pump activity to the generation of the neuronal rhythms are not fully understood.

We studied single-cell dynamics in endogenously bursting interneurons (HN neurons) from the leech heartbeat CPG. This CPG is comprised of pairs of HN neurons distributed along ventral nerve cord ganglia. HN neurons in ganglia 3, 4, and 7 have the same voltage-gated currents and behave as endogenous bursters when recorded extracellularly but lose rhythmicity when recorded with sharp microelectrodes (Cymbalyuk et al., 2002). HN neurons have been well characterized through numerous voltage-clamp studies (Angstadt and Calabrese, 1989, 1991; Simon et al., 1992; Opdyke and Calabrese, 1994; Tobin and Calabrese, 2005; Kueh et al., 2016), a biophysically accurate single compartment model has been constructed (A.A. Hill et al., 2001; A.A.V. Hill et al., 2002; Kueh et al., 2016; Ellingson et al., 2021) and rigorously tested experimentally (Sorensen et al., 2002; Olypher et al., 2006; Kueh et al., 2016; Ellingson et al., 2021; Erazo-Toscano et al., 2021), and special roles of the persistent Na^+^ and pump currents identified (Opdyke and Calabrese, 1994; A.A. Hill et al., 2001; Tobin and Calabrese, 2005; Doloc-Mihu and Calabrese, 2014; Kueh et al., 2016; Ellingson et al., 2021; Erazo-Toscano et al., 2021), altogether making HN neurons an ideal candidate for studying the mechanisms of rhythmic bursting and the role of the Na^+^/K^+^ pump.

The persistent Na^+^ current (I_NaP_) is a noninactivating voltage-gated inward current found in HN neurons; I_NaP_ supports burst formation and controls spike frequency (Opdyke and Calabrese, 1994). I_NaP_ is a target of neuromodulation by neuropeptide FMRFamide and the rhythmicity of HN neurons is highly sensitive to I_NaP_. For example, low concentrations of FMRFamide speed up the burst rhythm of HN neurons, while high concentrations disrupt neural activity (Simon et al., 1992; Schmidt et al., 1995; Nadim and Calabrese, 1997). I_NaP_ must be balanced by an outward current such as noninactivating K^+^ current_,_ leak current [Doloc-Mihu and Calabrese, 2014; e.g., the HN persistent K^+^ current (I_K2_); see Materials and Methods], or I_pump_. However, the dynamics that arise from the interaction between I_NaP_ and either I_K2_ or I_pump_ are fundamentally different because I_K2_ represents a typical conductance-based voltage-activated current while I_pump_ is a voltage-independent Na^+^-activated current.

Computational modeling and real-time hybrid systems such as the dynamic clamp are essential tools to unravel neuronal dynamics. We implemented a dynamic clamp to inject I_NaP_ and I_pump_ into single HN neurons based on a published computational model (A.A. Hill et al., 2001; Cymbalyuk et al., 2002; A.A.V. Hill et al., 2002; Olypher et al., 2006; Barnett and Cymbalyuk, 2011; Kueh et al., 2016; Ellingson et al., 2021; Erazo-Toscano et al., 2021). Our methods allow us to investigate how the interaction of these two ionic currents affects the rhythmic activity of isolated HN neurons. Our experiments with the HN neurons revealed a new bursting regime distinguished by a large amplitude of voltage oscillations and high spike frequency (HA bursting), elicited by the interaction of I_NaP_ and I_pump_. We determined that increasing I_pump_ simultaneously decreased burst duration (BD) and interburst interval (IBI) of this bursting regime. We have developed a reduced computational model that explains the mechanism underlying the speeding up of the neuronal rhythm. We demonstrate that the interaction of I_NaP_ and I_pump_ creates a flexible and robust oscillatory mechanism that supports the HA bursting.

## Materials and Methods

### Animals, solutions, and preparation

Leeches *Hirudo verbana* (https://www.leech.com/collections/live-leeches) were kept in artificial pond water [0.05% w/v Instant Ocean Sea (Spectrum Brands Inc.) diluted in deionized water] at 16°C on a 12/12 h light/dark cycle. Before each dissection, the animals were immobilized by burying them in a bed of crushed ice. For the dissection, the animal was immersed in a dissecting dish filled with chilled leech saline containing 115 mM NaCl, 4 mM KCl, 1.7 mM CaCl_2_, 10 mM D-glucose, and 10 mM HEPES in deionized water; pH adjusted to 7.4 with 1 M NaOH. The animals were pinned dorsal side up, and a longitudinal incision cut to expose the internal organs. Individual mid-body ganglia 7 were removed from the animal and pinned ventral side up in a 35-mm sylgard-coated (Sylgard, Dow Corning) Petri dish and covered with saline. Directly before the experiment, the glial sheath was removed from the ganglion with microscissors and a scalpel. The preparation was superfused continuously with leech saline (bath volume ∼0.5 ml, flow rate ∼5 ml/min). All experiments were performed at room temperature (21–22°C).

### Electrophysiology

Electrodes were pulled on a Flaming/Brown micropipette puller (P-97, Sutter Instruments) from borosilicate glass (1 mm OD, 0.75 mm ID; A-M Systems). The glass electrodes were filled with 2 M KAcetate and 20 mM KCl; we performed quality control to select only electrodes with a resistance higher than 30 MΩ. All intracellular traces were acquired using an Axoclamp 2A amplifier (Molecular Devices) in discontinuous current-clamp mode (DCC) at a switching/sampling rate >3 kHz. Intracellular signals were digitized with an Axon Digidata 1440A digitizer (5-kHz acquisition rate) and recorded with the Clampex 10.4 software (Molecular Devices).

After impaling the neuron with the sharp microelectrode, we injected a steady negative current (−0.3 nA) into the neuron for ∼3 min, then progressively removed the injected bias current in steps of 0.01 nA until the magnitude of the injected current was reduced to −0.1 nA; the neuron was kept with −0.1-nA steady current for the remainder of the experiment.

### Dynamic clamp

HN neurons have been extensively studied using voltage clamp, and the following currents were described: a fast Na^+^ current (I_NaF_), a persistent Na^+^ current (I_NaP_; Opdyke and Calabrese, 1994), a hyperpolarization-activated inward current (I_h_; Angstadt and Calabrese, 1989), and three K^+^ currents (Simon et al., 1992), delayed rectifier-like K^+^ current (I_K1_), a persistent K^+^ current (I_K2_), and a fast transient K^+^ current (I_KA_). For each of these currents model, Hodgkin-Huxley style equations have been developed (A.A. Hill et al., 2001; A.A.V. Hill et al., 2002; Kueh et al., 2016; Ellingson et al., 2021; Erazo-Toscano et al., 2021). A pump current has also been isolated in voltage clamp (Tobin and Calabrese, 2005), characterized, and model equations developed (Kueh et al., 2016). The dynamic clamp is implemented in MATLAB and Simulink (MathWorks) on dSpace real-time digital signal processing boards: the DS1104 R&D, and the DS1103 PPC (dSpace Inc.; Olypher et al., 2006; Barnett and Cymbalyuk, 2011). The dynamic clamp system executed the model HN persistent Na^+^ current (I_NaP_) and the model HN Na^+^/K^+^ pump current (I_pump_) equations so that these currents can be added to or subtracted from the recorded neuron. The dynamic clamp implementation of these currents has been described previously (Barnett and Cymbalyuk, 2011; Erazo-Toscano et al., 2021). Briefly, the dynamic clamp reads the membrane potential of the neuron from the Axoclamp 2A amplifier and updates the current injected by the amplifier according to model equations evaluated in real time (>3 kHz). The dynamic clamp also estimates the cell’s native fast Na^+^ current (spike-generating current; I_NaF_), native persistent Na^+^ current (I_NaP native_) and native pump current (I_pump native_) along with the injected I_NaP_ and I_pump_ to then estimate the intracellular Na^+^ concentration ([Na^+^]_i_) using Equation 1:

(1) $$\frac{d{\left[Na^{+}\right]}_{i}}{dt}=-\frac{I_{NaP}+I_{NaP\;native}+I_{NaF}+3I_{pump}+3I_{pump\;native}}{vF},$$

where vF (vF = 0.0024 nl × C/mol) is the volume ( ≈4.4 ρl) of the cytosolic Na^+^ reservoir multiplied by the Faraday constant (96,485 C/mol). The extracellular Na^+^ concentration ([Na^+^]_o_) is assumed to be constant. In the dynamic clamp model, we simplified the evaluation of Na^+^ dynamics and did not consider Na^+^ fluxes generated by the leak and h-currents (compare to Eq. 4).

The calculated intracellular ${\left[Na^{+}\right]}_{i}$ is used to compute the injected I_pump_ and to estimate native pump current:

(2) $$I_{pump}=\frac{I_{max}^{pump}}{1+e^{\left(\frac{{\left[Na^{+}\right]}_{ih}-{\left[Na^{+}\right]}_{i}}{{\left[Na^{+}\right]}_{is}}\right)}},$$

where $I_{max}^{pump}$, [Na^ + ^]_ih_, [Na^+^]_is_ determine the maximal value, the intracellular Na^+^ concentration for half-activation, and the sensitivity of the Na^+^/K^+^ pump current to [Na^+^]_i_, respectively. On the other hand, the injected I_NaP_ depends on the recorded HN neuron’s membrane potential and the evaluated [Na^+^]_i_, which determines Na^+^ reversal potential. In our dynamic-clamp experiments, the I_pump_ and I_NaP_ were controlled by $I_{max}^{pump}$ and ${\bar{g}}_{NaP}$, respectively; and where noted, we estimated the native currents I_NaP native_ ( ${\bar{g}}_{NaP\;native}$ = 5 nS) and I_pump native_ ( $I_{max\;native}^{pump}$= 0.1 nA), which were assumed to be endogenously present in HN neurons.

Dynamic clamp model is described by the following system of ordinary differential equations with the parameters provided in Table 1 with the steady state activation and inactivation functions described by the Boltzmann function $f_{\infty }\left(A,\;B,\;V\right)=\frac{1}{1+e^{A\cdot \left(V-B\right)}}$ .

$$C_{m}\;\frac{dV}{dt}\;=\;-(I_{NaF}+I_{NaP}+I_{NaP\;native}+I_{K1}+I_{K2}+I_{KA}+I_{h}+I_{CaF}+I_{CaS}+I_{pump}+I_{leak})$$

$$I_{NaF}={\bar{g}}_{NaF}\cdot m_{NaF}^{3}\cdot {\text{h}}_{NaF}\cdot \left(V-E_{Na}\right)$$

$$I_{NaP}={\bar{g}}_{NaP}\cdot m_{NaP}\cdot \left(V-E_{Na}\right)$$

$$I_{NaP\;native}={\bar{g}}_{NaP\;native}\cdot m_{NaP\;native}\cdot \left(V-E_{Na}\right)$$

$$I_{K1}={\bar{g}}_{K1}\cdot m_{K1}^{2}\cdot h_{K1}\cdot \left(V-E_{K}\right)$$

$$I_{K2}={\bar{g}}_{K2}\cdot m_{K2}^{2}\cdot \left(V-E_{K}\right)$$

$$I_{KA}={\bar{g}}_{KA}\cdot m_{KA}^{2}\cdot h_{KA}\cdot \left(V-E_{K}\right)$$

$$I_{h}={\bar{g}}_{h}\cdot m_{h}^{2}\cdot \left(V-E_{h}\right)$$

$$I_{CaF}={\bar{g}}_{CaF}\cdot m_{CaF}^{2}\cdot h_{CaF}\cdot \left(V-E_{Ca}\right)$$

$$I_{CaS}={\bar{g}}_{CaS}\cdot m_{CaS}^{2}\cdot h_{CaS}\cdot \left(V-E_{Ca}\right)$$

$$I_{pump}=\frac{I_{max}^{pump}}{1+e^{\frac{{\left[Na^{+}\right]}_{ih}-{\left[Na^{+}\right]}_{i}}{{\left[Na^{+}\right]}_{is}}}}$$

$$I_{leak}=g_{leak}\cdot \left(V-E_{leak}\right)$$

$$\frac{dm_{NaF}}{dt}=\frac{f_{\infty }\left(-150,\;0.029,\;V\right)-m_{NaF}}{0.0001}$$

$$\frac{dh_{NaF}}{dt}=\frac{f_{\infty }\left(500,\;0.03,\;V\right)-h_{NaF}}{{\text{τ}}_{hNa}(V)}$$

$${\text{τ}}_{hNa}(V)=0.004+\frac{0.02}{cosh\left[300\cdot \left(V+0.0027\right)\right]}+\frac{0.006}{1+e^{\left(500\cdot \left(V+0.028\right)\right)}}$$

$$\frac{dm_{NaP}}{dt}=\frac{f_{\infty }\left(-120,\;0.039,\;V\right)-m_{NaP}}{\text{τ}\left(400,\;0.057,\;0.01,\;0.2,V\right)}$$

$$\frac{dm_{NaP\;native}}{dt}=\frac{f_{\infty }\left(-120,\;0.039,\;V\right)-m_{NaP\;native}}{\text{τ}\left(400,\;0.057,\;0.01,\;0.2,V\right)}$$

$$\frac{dm_{K1}}{dt}=\frac{f_{\infty }\left(-143,\;0.021,\;V\right)-m_{K1}}{\text{τ}\left(150,\;0.016,\;0.001,\;0.011,V\right)}$$

$$\frac{dh_{K1}}{dt}=\frac{f_{\infty }\left(111,\;0.028,\;V\right)-h_{K1}}{\text{τ}\left(-143,\;0.013,\;0.5,\;0.2,V\right)}$$

$$\frac{dm_{K2}}{dt}=\frac{f_{\infty }\left(-83,\;0.02,\;V\right)-m_{K2}}{\text{τ}\left(200,\;0.035,\;0.057,\;0.043,V\right)}$$

$$\frac{dm_{KA}}{dt}=\frac{f_{\infty }\left(-130,\;0.044,\;V\right)-m_{KA}}{\text{τ}\left(200,\;0.03,\;0.005,\;0.011,V\right)}$$

$$\frac{dh_{KA}}{dt}=\frac{f_{\infty }\left(160,\;0.063,\;V\right)-h_{KA}}{\text{τ}\left(-300,\;0.055,\;0.026,\;0.0085,V\right)}$$

$$\frac{dm_{h}}{dt}=\frac{f_{\text{h}\infty }\left(V\right)-m_{h}}{\text{τ}\left(-100,\;0.073,\;0.7,\;1.7,V\right)}f_{h\infty }\left(V\right)=\frac{1}{1+2\cdot e^{180\cdot \left(V+0.047\right)}+e^{500\cdot \left(V+0.047\right)}}$$

$$\frac{dm_{CaF}}{dt}=\frac{f_{\infty }\left(-600,\;0.0467,\;V\right)-m_{CaF}}{\text{τ}\left(-330,\;0.0467,0.011,\;0.024,\;V\right)}$$

$$\frac{dh_{CaF}}{dt}=\frac{f_{\infty }\left(350,\;0.055,\;V\right)-h_{CaF}}{\text{τ}\left(270,\;0.055,\;0.06,\;0.31,V\right)}$$

$$\frac{dm_{CaS}}{dt}=\frac{f_{\infty }\left(-420,\;0.0472,\right)-m_{CaS}}{\text{τ}\left(-400,\;0.0487,\;0.005,\;0.134,V\right)}$$

$$\frac{dh_{CaS}}{dt}=\frac{f_{\infty }\left(360,\;0.055,\right)-h_{CaS}}{\text{τ}\left(-250,\;0.043,\;0.2,\;5.25,V\right)}$$

$$E_{Na}=0.02526ln\left(\frac{{\left[Na^{+}\right]}_{o}}{{\left[Na^{+}\right]}_{i}}\right).$$

**Table 1:** Parameters of dynamic clamp model

Parameter	Value	Parameter	Value
${\bar{g}}_{NaF}$	150 nS	$I_{pump}^{max}$	[0.3, 0.9] nA
${\bar{g}}_{NaP}$	[1, 6] nS	Na_ih_	0.012 M
${\bar{g}}_{NaP-native}$	5 nS	Na_is_	0.0016 M
${\bar{g}}_{K1}$	80 nS	Na_o_	0.115 M
${\bar{g}}_{K2}$	80 nS	E_K_	−0.07 V
${\bar{g}}_{KA}$	100 nS	E_h_	−0.021 V
${\bar{g}}_{h}$	4 nS	E_Ca_	0.135 V
${\bar{g}}_{CaF}$	5 nS	E_leak_	−0.045 V
${\bar{g}}_{CaS}$	4 nS		
$g_{leak}$	7 nS		

### Data analysis of experimental burst characteristics

**Figure 1.**
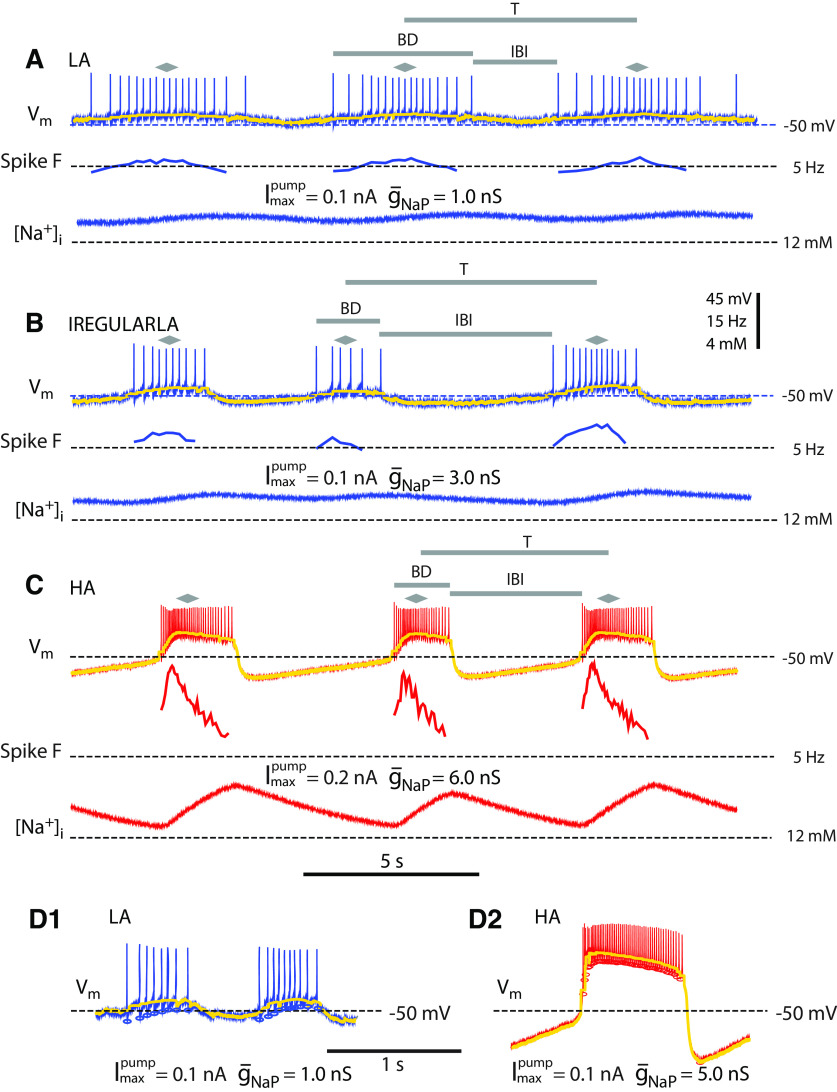
Experimental data from a single HN(7) neuron with I_NaP_ and I_pump_ introduced by dynamic clamp to support bursting activity. In ***A–C***, membrane potential V_m_ (mV; top), the instantaneous spike frequency (Hz; middle), and virtual internal Na^+^ concentration ([Na^+^]_i_; mM; bottom) estimated by the dynamic clamp model. In this and all subsequent figures [Na^+^]_i_ is calculated using both estimated native (I_pump_, I_NaP_, and I_NaF_) and dynamic clamp injected (I_pump_ and I_NaP_) currents. Parameter values for ${\bar{g}}_{NaP}$ and $I_{max}^{pump}$ are those used for dynamic clamp injected currents. The horizontal bars above the membrane potential traces mark the temporal characteristics of the bursting: bursting cycle period (T), the interburst interval (IBI), and the burst duration (BD). The gray diamonds over each burst tag the median spike used to compute the burst cycle period. The thick colored curves over the voltage traces are the (voltage) envelopes of burst waveforms (Materials and Methods). In this and subsequent figures, high voltage amplitude (HA), high spike frequency bursts are shown in red with the corresponding voltage envelope in yellow, and low voltage amplitude (LA), low spike frequency bursts are shown in blue with the corresponding voltage envelope in yellow as well. ***A–C***, Sample data for different dynamic clamp ${\bar{g}}_{NaP}$ and $I_{max}^{pump}$ parameters from the same preparation. ***A***, Coefficient of variation for cycle period: 0.25. ***B***, Coefficient of variation for cycle period: 0.38. ***C***, Coefficient of variation for cycle period: 0.1. ***D1***, ***D2***, Computed voltage envelope with spike minima identified by ovals. These are representative bursts, LA (***D1***) and HA (***D2***), respectively, with different dynamic clamp parameters.

We computed the experimental burst characteristics using previously described methods (Masino and Calabrese, 2002). We detected spikes in the membrane potential traces with a Matlab function identifying the peaks and selecting those with a height larger than 20 mV. First, we computed the interspike intervals (ISIs) and the ISIs larger than 800 ms were discriminated as the interburst intervals (IBIs). The burst duration (BD) was the time between the first and last spikes of the burst (Fig. 1), where a burst was defined as a group of five or more spikes. A group with a smaller number of spikes was discarded and its time interval was concatenated with the two large ISIs, surrounding this small group, into a single interburst interval. The spiking activity within bursts was characterized by the total number of spikes, instantaneous spike frequency defined for each spike as 1/ISI of the interval following its peak, and the average intraburst spike frequency which for short we call spike frequency. The interburst interval (IBI) was the time between the last spike of a burst and the first spike potential of the subsequent burst (Fig. 1). The IBI was assigned to the preceding burst. The cycle period (T), used to assess rhythm regularity, was defined as the time difference between the median spikes of two subsequent bursts (Fig. 1) and was assigned to the first of the two bursts. We required a minimum of eight consecutive bursts that could be detected for a sample recording to be accepted into the analysis.

We characterized a spiking-silence waveform for a bursting cycle by a basic envelope of the membrane potential oscillation with depolarized and hyperpolarized phases. For the depolarized phase, we averaged membrane potential (V_m_) over the time interval between the consecutive minima bounding each spike and assigned the value obtained for every point within this interval. Between bursts, V_m_ was harvested as is, without averaging. We used the envelope of the bursting cycle to compare membrane potential waveforms obtained for different parameter sets and to compare with the simple oscillatory waveform of a two-dimensional model describing interaction of the membrane potential and intracellular Na^+^ concentration (Fig. 1*D*).

We computed the amplitudes of the averaged V_m_, [Na^+^]_i_, and I_pump_ oscillations of the bursting envelope by detecting the peaks and troughs of the signal and then subtracting the trough values from the peak values in a pairwise fashion. We also computed the median [Na^+^]_i_ and I_pump_, as the median value of the signal between peaks and troughs of the [Na^+^]_i_ and I_pump_, respectively; thus, the median was computed for the backside of the waveform (from maximum to minimum), only. We reported the averaged median across recorded cycles of oscillations.

In summary, we gathered the following characteristics of bursting activity: burst duration (BD), interburst interval (IBI), cycle period (T), amplitude of the voltage envelope, instantaneous spike frequency, spike frequency, [Na^+^]_i_ envelope amplitude, median [Na^+^]_i_, I_pump_ envelope amplitude, and median I_pump_. We performed normalization by dividing the dependent variables (DVs) of interest (BD, IBI, [Na^+^]_i_ and I_pump_ amplitude values, [Na^+^]_i_ and I_pump_ median values) by the value of the corresponding DV at $I_{max}^{pump}$ = 0.3 nA, ${\bar{g}}_{\mathrm{NaP}}$ = 6 nS. These reference values of the parameters were chosen since this combination consistently supported regular rhythmic bursting. Normalization was performed within each individual.

### Machine learning algorithms

We implemented a two-threshold algorithm to discriminate between low and high-amplitude oscillations of the membrane potential and [Na^+^]_i_ in the dynamic clamp experimental data (Fig. 1*D1*,*D2*). An expert assisted in discriminating the two regimes with the two-threshold algorithm on 35% of the randomly selected data points by choosing the thresholds. As the result, the classification algorithm assigned a value of “1” to the neuron sample activity if both the membrane potential amplitude and intracellular sodium concentration amplitude were larger than the respective thresholds of 20 mV and 1 mm, indicating high-amplitude bursting activity. Conversely, a value of “0” was assigned if either or both of the amplitudes were below or equal to their respective thresholds, indicating low amplitude bursting activity. The output of the two-threshold algorithm was used to train a Gaussian naive Bayes supervised machine learning (ML) algorithm, which then was applied to classify the remaining 65% of the data. The ML algorithm was implemented in Python 3.6.8 with the Scikit-learn 1.1.3 library obtained from the Anaconda distribution; and further details about the naive Bayes algorithm can be found in the scikit-learn documentation (https://scikit-learn.org/stable/modules/naive_bayes.html).

**Figure 2.**
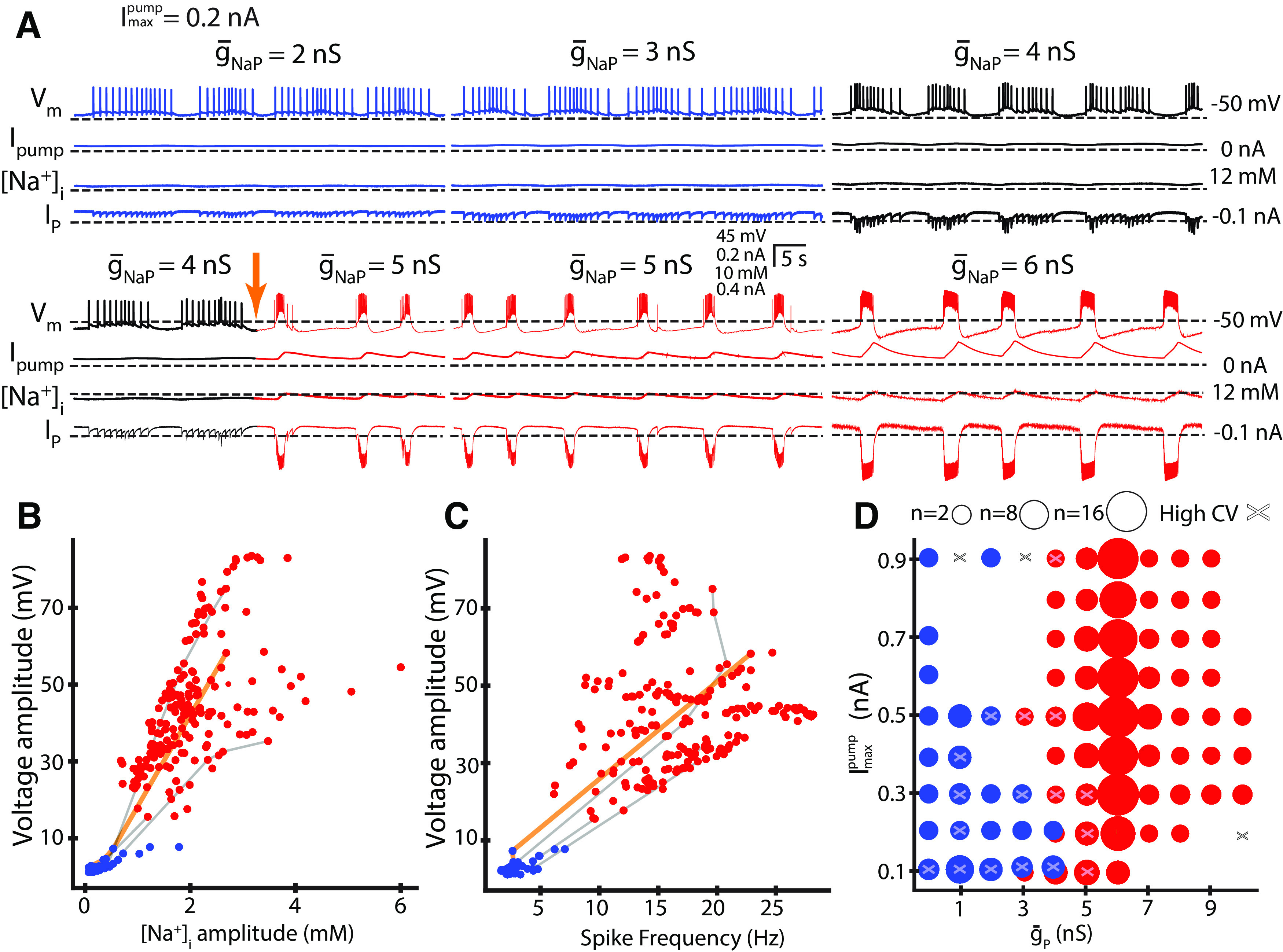
Two bursting regimes observed while varying ${\bar{g}}_{\mathrm{NaP}}$ and $I_{max}^{pump}$ introduced by dynamic clamp. ***A***, This set of voltage traces exemplify how while keeping $I_{max}^{pump}$ at 0.2 nA and setting ${\bar{g}}_{NaP}$ above some critical value, causes a transition of the neuron into the HA bursting regime. In this and all subsequent figures I_pump_ and I_NaP_ are dynamic-clamp injected currents and ${\bar{g}}_{NaP}$ and $I_{max}^{pump}$ are their parameter values, respectively. The voltage traces are from the same preparation under similar conditions; the only thing that changes between subsequent traces is the ${\bar{g}}_{P}$ parameter, the arrow points at the moment when the experimenter changed ${\bar{g}}_{NaP}$ from 4 to 5 nS. ***B***, Scatterplot of waveform envelope voltage amplitude and [Na^+^]_i_ amplitude. ***C***, Scatterplot of voltage amplitude and average intraburst spike frequency. ***D***, Two-parameter map ( ${\bar{g}}_{NaP\;}\;I_{max}^{pump}$) of color-codes parameter sets exhibiting two different bursting regimes: the LA and HA bursting regimes are separated to the left and right areas of the map, blue and red groups, respectively. There is an area where blue and red data points overlap at the border. The size of each circle indicated the number of accepted experiments with the color-coded outcome. x indicates that there were experiments with high variability in cycle period T (high coefficient of variation: *K* ≥ 0.27) that were excluded with this parameter set from the analysis. The threshold for the transition from the LA to the HA bursting varies between neurons and may result from animal-to-animal variability (Wenning et al., 2018). ***B–D***, Output from Gaussian naive Bayes machine learning classification algorithm are color-coded by red and blue; red for the HA bursting and blue for the LA rhythm. Gray lines connect data points from the same experiment (voltage traces not shown). The gold line connects the data points from the sample traces (***A***). Data points are connected in ascending ${\bar{g}}_{NaP}$ order, not in the order the parameters were varied. In most experiments, we varied ${\bar{g}}_{NaP}$ in a random order, but for illustration purposes, the parameters for the experimental traces in ***A*** are shown in ascending order.

We performed linear regression analyses between the amplitude of voltage bursting envelope (voltage amplitude) and [Na^+^]_i_ envelope amplitude and between the voltage amplitude and spike frequency; the regression analyses were performed in two complementary ways. First, we computed collinearity in an experiment-by-experiment fashion. Second, we computed collinearity on the pooled data. All statistical analyses, including linear regression and nonparametric Mann–Whitney *U* test were performed using Python 3.6.8 and Scipy 1.8 stats from the Anaconda distribution.

### Simplified HN neuron model

We propose that the interaction between I_NaP_ and I_pump_ can explain the dynamical mechanism underlying the phenomena we observed experimentally and have developed a simplified version of the HN model focused on this interaction in the canonical HN model (A.A. Hill et al., 2001; A.A.V. Hill et al., 2002; Kueh et al., 2016; Ellingson et al., 2021; Erazo-Toscano et al., 2021). It has two state variables: membrane potential, V_m_, and intracellular Na^+^ concentration, ${\left[Na^{+}\right]}_{i}$. The membrane potential variable governs four voltage-gated currents with instantaneous activation/inactivation: persistent sodium current with the two components, I_NaP_ and I_NaP native_, fast sodium current (I_NaF_), hyperpolarization-activated current (I_h_), and noninactivating potassium current (I_K2_). The intracellular Na^+^ concentration variable governs the pump current (I_pump_) and modulates Na^+^ currents through the changes in Na^+^ reversal potential. Leak and hyperpolarization-activated currents were split into K^+^ and Na^+^ components (I_leak-K_ and I_leak-Na_ and I_h-K_ and I_h-Na_). This model adopted the same parameters values of the two injected currents I_NaP_ and I_pump_ used in experiments. The equations of the simplified model are as follows:

(3) $$\frac{dV_{m\;}}{dt}=-\frac{1}{C_{m}}\left(I_{NaP\;native}+I_{NaP\;}+I_{pump}+I_{NaF}+I_{h-Na}+I_{h-K}+I_{leak-Na}+I_{leak-K}+I_{K2}\right)$$

(4) $$\frac{d{\left[Na^{+}\right]}_{i}}{dt}=-\frac{I_{NaP\;native}+I_{NaP\;}+3I_{pump}+I_{NaF}+I_{h-Na}+I_{leak-Na}}{vF}.$$

Equations 3 and 4 estimate division between Na^+^ and K^+^ components of h-current as $I_{h-Na}=\frac{3}{7}I_{h}$ and $I_{h-K}=\frac{4}{7}I_{h}$ suggested by the h-current reversal potential −21 mV (Kueh et al., 2016).

In contrast to the dynamic clamp, in the 2D model, the evaluation of Na^+^ dynamics includes native Na^+^ currents in addition to dynamic clamp currents. The individual voltage-gated currents were computed with instantaneous activation and inactivation gating variables:

$$I_{i}={\bar{g}}_{i}\cdot m_{\infty i}^{a_{i}}(V_{m\;})\cdot h_{\infty i}^{b_{i}}(V_{m\;})\cdot \left(V_{m\;}-E_{i}\right),$$

where ${\bar{g}}_{i}$ and $E_{i}$ are the maximal conductance and reversal potential, respectively, with the subscript “*i*” identifying the type of the current (Table 2). The reversal potential for Na^+^ currents $E_{Na}$ was computed using the Nernst equation: $E_{Na}=\frac{RT}{zF}ln\frac{{\left[Na^{+}\right]}_{o}}{{\left[Na^{+}\right]}_{i}}$, where [Na^+^]_o_ is constant.

**Table 2:** Cell constants and current parameters: maximal conductance $\bar{g_{i}}$ **(nS), activation exponential a_i_, inactivation exponential b_i_, and reversal potential E_i_ (V)**

Cell constants	
C_m_	0.25 nF
Volume	0.0038 nl
[Na]_o_	0.115 M

Current parameters	
Current	${\bar{g}}_{i}$ (nS)	a_i_	b_i_	E_i_ (V)

$I_{NaP-native}$	9.85	1	0	E_Na_
$I_{NaP}$	6	1	0	E_Na_
$I_{NaF}$	150	3	1	E_Na_
$I_{h}$	6	2	0	−0.021
$I_{K2}$	138	2	0	−0.07

The voltage-gated activation and inactivation are instantaneous and denoted as the steady-state functions $m_{\infty i}(V_{m\;})$ and $h_{\infty i}(V_{m\;})$, respectively (Table 3). I_pump_ is determined by Equation 2 with parameters from Table 4. I_leak-K_ and I_leak-Na_ are Ohmic currents (parameters in Table 4), determined by equation where i stands for either K or Na:

$$I_{leak-i}=g_{leak-i}\cdot \left(V_{m\;}-E_{i}\right).$$

**Table 3:** Equations for computing steady-state activation $m_{\mathrm{\infty i}}$**(V) and inactivation** $h_{\mathrm{\infty i}}$ **(V)**

Current: variable	Steady-state activation or inactivation functions
$I_{NaP-native}:m_{\infty }$( $V_{m\;}$)	$\frac{1}{1+e^{-160\cdot \left(V+0.0376\right)}}$
$I_{NaP}:m_{\infty }$ ( $V_{m\;}$)	$\frac{1}{1+e^{-120\cdot \left(V+0.04\right)}}$
$I_{NaF}:m_{\infty }$ ( $V_{m\;}$)	$\frac{1}{1+e^{-210\cdot \left(V+0.032\right)}}$
$I_{NaF}:h_{\infty }$ ( $V_{m\;}$)	$\frac{1}{1+e^{500\cdot \left(V+0.04\right)}}$
$I_{h}:m_{\infty }$ ( $V_{m\;}$)	$\frac{1}{1+2\cdot e^{184.14\cdot \left(V+0.057\right)}+e^{511.5\cdot \left(V+0.057\right)}}$
$I_{K2}:m_{\infty }$ ( $V_{m\;}$)	$\frac{1}{1+e^{-83\cdot \left(V+0.0285\right)}}$

**Table 4:** Parameters for special currents I_leak_ and I_pump_

	${\bar{g}}_{leak}$ (nS)	E_i_ (V)	
$I_{leak-Na}$	1.1	E_Na_	
$I_{leak-K}$	8.8	−0.07	

	$I_{max}^{pump}$ (nA)	${\left[Na^{+}\right]}_{is}$(M)	${\left[Na^{+}\right]}_{ih}$(M)

$I_{pump}$	Range [0.2–0.9]	0.0016	0.012

### Data analysis of the 2D model

The 2D HN interneuron model produces a simple oscillatory voltage waveform similar to the experimental membrane potential envelope waveform. To divide the cycle period into the phases corresponding to the bursting waveform, we set a threshold at −45 mV and considered the depolarized and hyperpolarized phases as corresponding to the burst and silent phases, respectively following the definition of the phases established previously (Ellingson et al., 2021). Thus, in the 2D model simulated oscillations, BD is the time interval the voltage is above the threshold, and the IBI is the time interval when it is below the threshold. To compute the simulated amplitudes of V_m_ and [Na^+^]_i_ we subtracted the troughs from the peaks of the signal.

### Optimization of the simplified HN model

We used an evolutionary algorithm to find a 2D model parameter set that fits the envelop waveforms and temporal characteristics of bursting activity of an experimental set of used values of $I_{max}^{pump}$. The evolutionary algorithm optimized the parameters of native currents representing a given recorded living neuron: maximal conductance of native I_h_ – ${\bar{g}}_{h}$, the voltage of half-activation (V_½_) of I_h_, conductances of K^+^ and Na^+^ components of leak current, g_leak-K_ and g_leak-Na_, respectively, maximal conductance, steepness of activation, and voltage of half-activation of the native persistent Na^+^ current, and the maximal conductance of persistent K^+^ current – ${\bar{g}}_{K2}$. The algorithm kept the dynamic-clamp currents (injected I_NaP_ and I_pump_) parameters intact. To assess the quality of the model fit, we introduced a cost function that took into account the differences in temporal characteristics and deviations in maxima and minima of membrane potential and [Na^+^]_i_ waveforms between the experimental and model activities.

(5) $$F_{cost}=\sum^{}\left[{\left(BD_{exp}-BD_{sim}\right)}^{2}+{\left(IBI_{exp}-IBI_{sim}\right)}^{2}+\frac{{\left(V\;peak_{exp}-V\;peak_{sim}\right)}^{2}+{\left(V\;trough_{exp}-V\;trough_{sim}\right)}^{2}}{2}+\frac{{\left({\left[Na^{+}\right]}_{i}\;peak_{exp}-{\left[Na^{+}\right]}_{i}\;peak_{sim}\right)}^{2}+{\left({\left[Na^{+}\right]}_{i}\;\;trough_{exp}-{\left[Na^{+}\right]}_{i}\;\;trough_{sim}\right)}^{2}}{2}\right].$$

The evolutionary algorithm generated a population of an “f” number of parameter sets randomly generated around an initial canonical set. We also refer to these parameter sets as individuals. The outcome of the cost function evaluated all individuals, and the parameter set with the smallest value of the function was passed on to create the next generation. At the next generation, a population of individuals was created by adding small noise (2.5% of the parameter value) to each optimization parameter of the passed individual (Ashlock, 2006; Eiben, 2015). The algorithm iterated the process for “n” number of generations. We optimized the following targeted parameters: g_leak-Na_, g_leak-K_, ${\bar{g}}_{h}$, ${\bar{g}}_{NaF}$, native ${\bar{g}}_{NaP}$, the voltage of half-activation of native I_NaP-native_, steepness of activation of native I_NaP-native_, ${\bar{g}}_{K2}$ and the voltage of half-activation of I_K2_. These parameters represented cell to cell variability between preparations. The cost function incorporated the outcome of the experimental protocol and kept the experimental values of the parameters ${\bar{g}}_{NaP}$ and $I_{max}^{pump}$ the same while varying only the targeted parameters. An example of the code and analysis of a 2D model with a parameter set fitted to the bursting activity of an experimental set could be found in the Extended Data 1.

10.1523/ENEURO.0331-22.2023.ed1 Extended Data 1 The extended data compressed file contains the experimental data for an experimental set and the Matlab code for the 2D model fitted to the data. The file also provides scripts for the analysis of the experimental data and outcome of the model. The scripts are listed in ReadMeFirst.txt. Download Extended Data 1, ZIP file.

## Results

We investigated the dynamics emerging from the interaction between I_NaP_ and I_pump_ in isolated leech heart interneurons (HNs). We focused on the HN(7) interneurons because, it was essential for this study that in the seventh ganglion the two HNs do not have mutual synaptic connections and are thus isolated as soon as this ganglion is extracted. Thus, this preparation allows us to readily investigate single-cell dynamics without pharmacological intervention (Calabrese, 1977). We introduced the two currents with the dynamic clamp and used two different protocols to investigate the effect of varying ${\bar{g}}_{NaP}\;$ and $I_{max}^{pump}$ on activity of the interneuron. For the first protocol, we kept $I_{max}^{pump}$ constant at a value taken from the range [0.1–0.9 nA] (*N* = 40) and randomly varied ${\bar{g}}_{NaP}$ within the range between 1.0 and 7.0 nS. For the second protocol, we kept ${\bar{g}}_{NaP}$ constant at 6 nS (*N* = 13) and varied $I_{max}^{pump}$ within the range between 0.1 and 0.9 nA in random order. For each step in a protocol, we assessed whether the system had reached steady state by attainment of a [Na^+^]_i_ amplitude that showed small stochastic variability (Fig. 2*A*) and analyzed all cycles after this point requiring a minimum of eight for the data to be considered. Some animals were used for both protocols if the recorded interneuron remained functional for a sufficient length of time. In total, 41 animals were used for this study.

**Figure 3.**
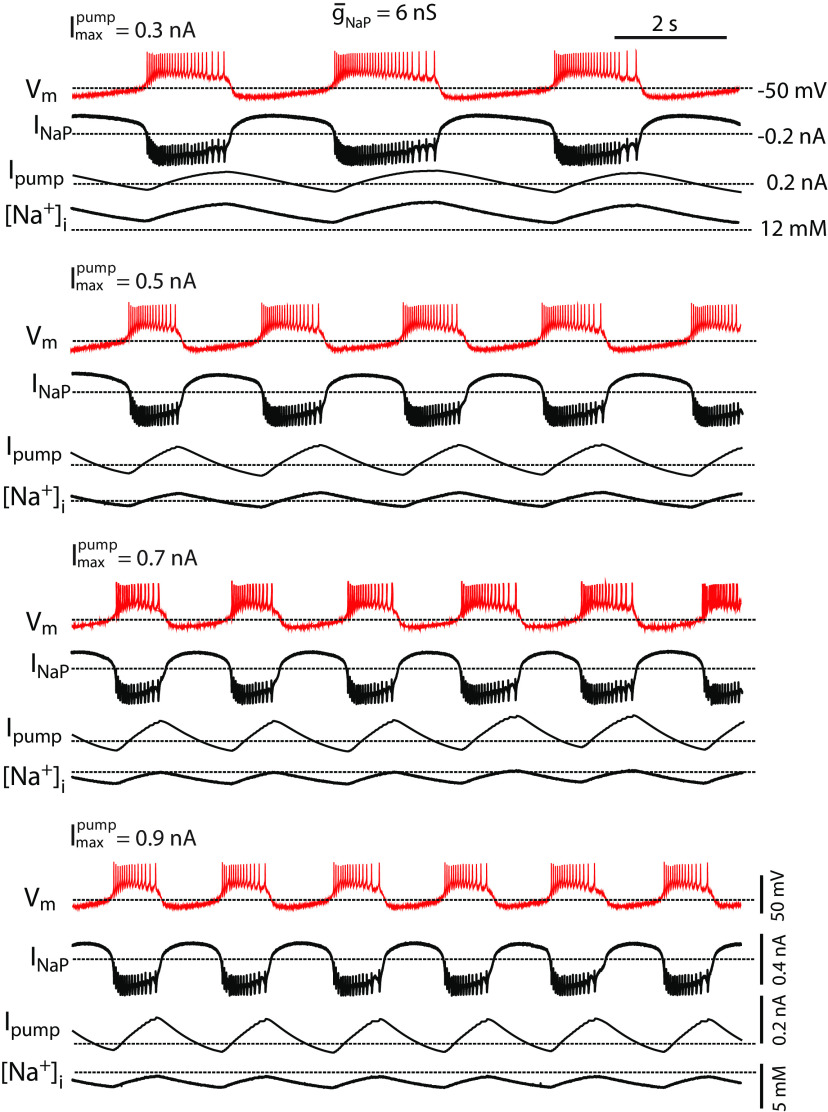
Increasing the maximal pump activity ( $I_{max}^{pump}$) speeds up the HA bursting regime. A set of dynamic clamp traces shows that while keeping the HN(7) neuron in the HA bursting regime with dynamic-clamp injected ${\bar{g}}_{NaP}=6$ nS, incrementing dynamic-clamp injected $I_{max}^{pump}$ decreased the burst duration and interburst interval, and thus the cycle period. Note that the increasing $I_{max}^{pump}$ changes the pump dynamics, by increasing the amplitude of the I_pump_ oscillation but not its median value. Each experimental data sample comprises a set of four signal traces recorded in real time by our dynamic clamp system, from top to bottom, the traces are membrane potential V_m_ (mV), injected I_NaP_ (nA), injected I_pump_ (nA), virtual [Na^+^]_i_ (mM).

### Injection of small I_NaP_ and I_pump_ reinstates bursting in HN neurons

The dynamics of a single HN are very sensitive to the shunting leak current introduced by sharp microelectrodes. The isolated HN interneurons exhibit bursting activity, if recorded extracellularly with a loose whole-cell patch pipette, and tonic spiking activity, if recorded intracellularly with a sharp microelectrode (Cymbalyuk et al., 2002). We found that we could reinstate bursting activity in HN neurons recorded with sharp microelectrodes by introducing relatively small currents, I_NaP_ ( ${\bar{g}}_{NaP}$ = 1 nS) and I_pump_ ( $I_{max}^{pump}$= 0.1 nA), using dynamic clamp. The reestablished bursting activity has properties typical for HN bursting: low voltage envelope amplitude underlying the slow membrane potential oscillations and the spike frequency similar to that obtained in intracellular recordings from neurons incorporated in a half-center oscillator (HCO; Fig. 1*A*; Tobin and Calabrese, 2005; Kueh et al., 2016). Below, we call this bursting regime with low amplitude voltage oscillations, LA bursting.

### The combination of $I_{max}^{pump}$ and ${\bar{g}}_{NaP}$ distinguishes two bursting regimes exhibited by hybrid HN neurons

Using the first dynamic clamp protocol, we systematically increased ${\bar{g}}_{NaP}$ while keeping $I_{max}^{pump}$ at a constant value (Fig. 1*A*,*B*,*D2*). When we increased ${\bar{g}}_{NaP}$ from 1.0 to 3.0 nS, the burst duration and interburst interval became irregular (Fig. 1*B*, coefficient of variation = 0.38). When we further increased ${\bar{g}}_{NaP}$ to 5 nS, the bursting activity became regular again and its waveform changed: the spike frequency and amplitude of the underlying envelope oscillations of V_m_ and [Na^+^]_i_ became visibly higher (Fig. 1*C*,*D2*). These observations suggested the emergence of a new bursting regime as a result of the variation of $I_{max}^{pump}$ and ${\bar{g}}_{NaP}$. Below, we call this regime the high-amplitude (HA) bursting.

We systematically varied dynamic-clamp parameters, $I_{max}^{pump}$ and ${\bar{g}}_{NaP},$ to test whether these two bursting regimes are quantitatively distinct. We found that the LA bursting possesses the following properties, the amplitude of membrane potential envelope had mean = 4.1 mV, SD = 2.1 mV (*n* = 15), spike frequency had mean = 4.1 Hz, SD = 1.4 Hz (*n* = 15), and estimated amplitude of [Na^+^]_i_ oscillation was relatively low with mean = 0.42 mm, SD = 0.14 mm (*n* = 15; Figs. 1*A*, 2*A*). The other regime, the HA bursting, has high amplitude of envelope of membrane potential with mean = 44.8 mV, SD = 1.16 mV (*n* = 22), high spike frequency with mean = 17.11 Hz, SD = 0.9 Hz (*n* = 22), and high amplitude of [Na^+^]_i_ oscillation with mean = 2 mm, SD = 0.13 mm (*n* = 22; Fig. 1*C*, 2*A*). For control conditions in HN(7) neurons, the average spike frequency in the literature is 11.4 ± 2.2 Hz (Cymbalyuk et al., 2002).

In all experiments, a sufficiently large increase of ${\bar{g}}_{NaP}$ caused the transition from the LA to HA bursting (Fig. 2). We also observed intermediate activities exhibiting mixed bursts with low and high-spike frequencies that resemble a hybrid of both HA and LA bursting (Figs. 1*B*, 2*A*, ${\bar{g}}_{NaP}$ = 4 nS, black traces). We discarded such activities along with any other activities with high variability of the cycle period (coefficient of variation ≥ 0.25) from further analysis. The decision to use this cutoff value was informed by the comparison to the whole population, which showed a low degree of variability in the burst cycle period (T). The coefficient of variation (CV) for the cycle period ranged from 0.01 to 0.43, with a median of 0.06 and first and third quantiles of 0.04 and 0.10, respectively. These statistics indicate that the majority of the population had a relatively low degree of variation. The burst duration CV showed a wider range of variation, spanning from 0.02 to 0.95, with a median of 0.11 and first and third quantiles of 0.07 and 0.17, respectively. Similarly, the interburst interval CV ranged from 0.02 to 0.79, with a median of 0.08 and first and third quantiles of 0.06 and 0.17. In HA bursting, I_NaP_ augmented the depolarization phase of the neuron, facilitated [Na^+^]_i_ influx, and supported increased spike frequency (Fig. 2*A*). The strong positive correlation between voltage envelope and [Na^+^]_i_ oscillation amplitudes demonstrate that membrane potential depolarization and [Na^+^]_i_ influx grow together (experiment-by-experiment averaged *r* = 0.98, *p* < 0.01, pooled data *r* = 0.766, *p* < 0.001, *n* = 22; Fig. 2*B*). The voltage amplitude and the spike frequency are also strongly positively correlated (experiment-by-experiment averaged *r* = 0.96, *p* ≤ 0.01, pooled data *r* = 0.83, *p* < 0.001, *n* = 22; Fig. 2*C*). The scatterplots (Fig. 2*B*,*C*) indicate the differences in the voltage amplitude, the intracellular Na^+^ concentration amplitude, and the spike frequency between the LA and HA bursting regimes. We used a two-threshold algorithm to classify our data and train a Gaussian naive Bayes supervised machine learning classification algorithm (ML; Materials and Methods). The quality of the classification is evaluated by a report table showing the accuracy of the classification in terms of precision and recall (Table 5). Precision is the ratio of true positives to the sum of true and false positives. The closer the precision score is to 1.0, the better the expected model performance. Recall is the ratio of true positives to the sum of true positives and false negatives. The closer the recall score is to 1.0, the better the expected model performance. The F1 score is the weighted harmonic mean of precision and recall. The closer the value of the F1 score is to 1.0, the better the expected performance of the model. Support is the number of actual occurrences of the class in the dataset, it diagnoses the performance evaluation process.

**Table 5:** Machine learning classification report

	Precision	Recall	F1-score	support
LA	1	0.92	0.96	12
HA	0.99	1	0.99	91
				
Accuracy			0.99	103
macro avg	0.99	0.96	0.98	103
weighted avg	0.99	0.99	0.99	103

Looking at the precision and recall scores, one can see that for both classes (LA and HA), the precision score is above 0.9 and the recall score is above 0.9, which indicates that the model is accurately identifying both classes with high precision and recall. The F1-score, which is a weighted average of precision and recall, is also very high for both classes, indicating a high level of overall accuracy. In addition, the support value for each class is relatively high, indicating that the model was tested on a sufficient amount of data to evaluate its performance accurately. Overall, the weighted average F1-score of 0.99 indicates that the classification model is performing at a very high level of accuracy, and the accuracy of 0.99 further supports this conclusion.

To test the accuracy of the ML classification algorithm and evaluate the meaningfulness of the separation between the LA and HA groups, we performed a one-tailed independent samples *t* test. The results showed a highly significant difference between the LA and HA groups (*t* = −13.38, *p* < 0.001, *n* = 44), indicating that the algorithm was successful in accurately distinguishing between these two bursting patterns.

We mapped the LA and HA bursting regimes distinguished by the classification algorithm on the parameter plane ( ${\bar{g}}_{P},\;I_{max}^{pump}$; Fig. 2*D*). While the LA bursting clusters at the bottom left corner of the scatterplots, the HA rhythm spreads out on the upper area of the plots (Fig. 2*B*,*C*). A two-parameter map of ${\bar{g}}_{\mathrm{NaP}}$ and $I_{max}^{pump}$ shows that ${\bar{g}}_{P}$ determines the switch between LA and the HA bursting (Fig. 2*D*). LA and HA bursting are segregated to different areas where ${\bar{g}}_{\mathrm{NaP}}$ is lower (LA) or higher (HA). Because of animal-to-animal variability, there is also an area of overlap where both regimes were observed.

### In the HA bursting, increase of $I_{max}^{pump}$ decreases HN burst duration and interburst interval

The Na^+^/K^+^ pump current is voltage-independent and can be activated during and between bursts. Because the Na^+^/K^+^ pump current can thus regulate neuronal activity throughout the burst cycle, it potentially affects both BD and IBI. The Na^+^/K^+^ pump current is activated by increased [Na^+^]_i_. In both the LA and HA bursting regimes, [Na^+^]_i_ increases during bursts, but in the HA, the [Na^+^]_i_ reaches higher values because of the increased joint contribution of injected I_NaP_ and estimated I_NaF_ (Fig. 1). Then, the Na^+^/K^+^ pump remains active after the burst termination throughout the IBI. The interaction between I_pump_ and I_NaP_ controls the IBI by providing mutual feedback. Because of its [Na^+^]_i_-dependence, the Na^+^/K^+^ pump responds to an increase in intracellular sodium concentration ([Na^+^]_i_) and could terminate bursts. Demonstrating that the peaks of the oscillations of I_pump_ coincide with the last spike of the burst, Figure 3 agrees with this mechanism of burst termination. When [Na^+^]_i_ declines, the I_pump_ decreases, permitting I_NaP_ to depolarize the membrane potential and initiate a new cycle (Fig. 3). The troughs of the oscillations of I_pump_ coincide with the first spike of each burst.

**Figure 4.**
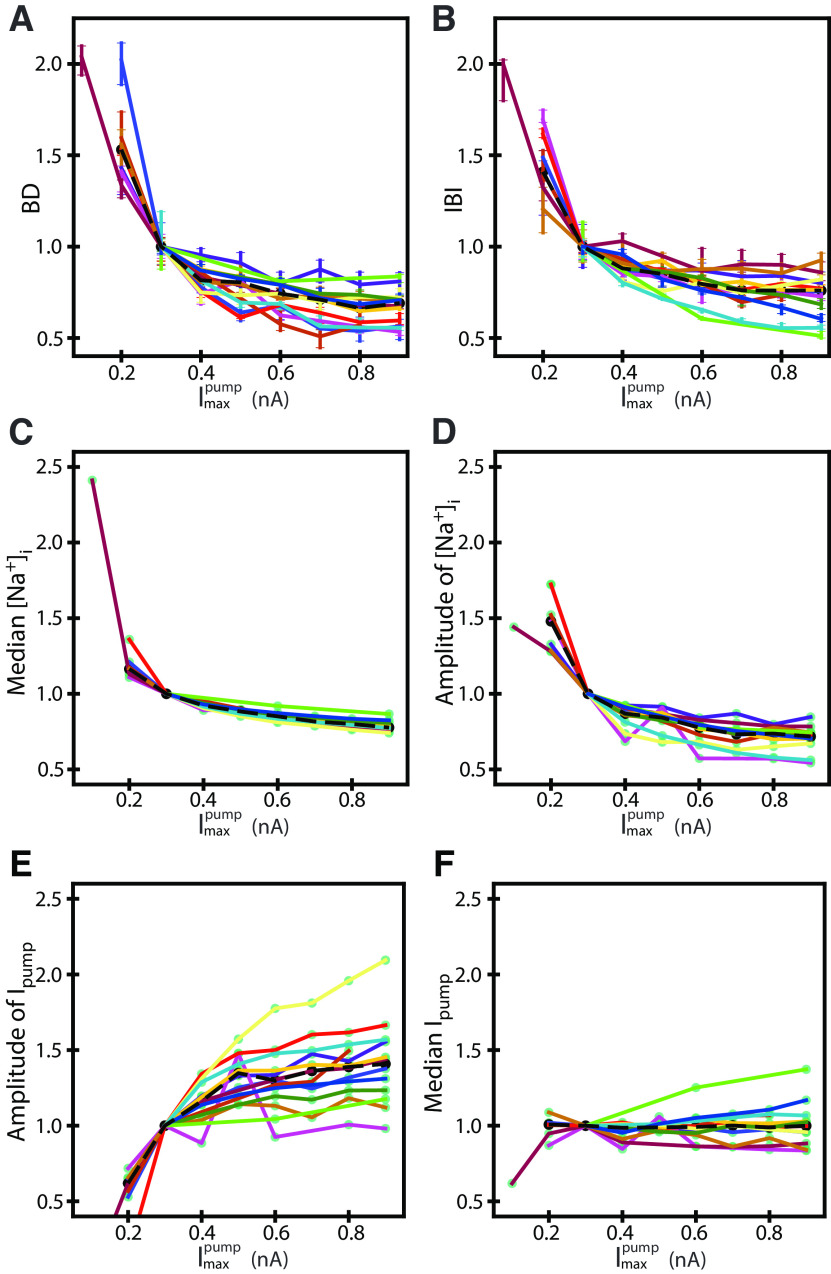
Increasing dynamic-clamp injected $I_{max}^{pump}$ decreased burst duration, interburst interval, and [Na^+^]_i_ in the HA bursting regime. The average trend across experiments in the HA bursting regime shows that increasing dynamic-clamp injected $I_{max}^{pump}$ decreases the following characteristics, burst duration (***A***) and interburst interval (***B***), the median [Na^+^]_i_ (***C***), and [Na^+^]_i_ amplitude (***D***). However, increasing $I_{max}^{pump}$ does not affect the median I_pump_ (***E***) but increases the amplitude of the I_pump_ oscillations (***F***). Changes of different characteristics are shown as normalized pooled data from multiple experiments, represented by different colors consistent between subplots. The black line consists of the mean value across experiments for a given $I_{max}^{pump}$.

In the HA regime, increasing $I_{max}^{pump}$ speeds up the period of HN bursting (Figs. 3, 4*A*,*B*). Since the parameter $I_{max}^{pump}$ describes the maximal output of the I_pump_ but does not affect the pump’s sensitivity or the concentration of half-activation of I_pump_, $I_{max}^{pump}$ determines [Na^+^]_i_ and I_pump_. Namely, an increase of $I_{max}^{pump}$ from 0.3 to 0.7 nA decreased the median [Na^+^]_i_ by 18% [for $I_{max}^{pump}$= 0.3 nA, 90% confidence interval (CI): 12.59 13.35, range: 11.69–14.37, *n* = 13; for $I_{max}^{pump}$= 0.7 nA, 90% CI: 10.32–10.86, range: 9.86–11.38, *n* = 13] and the [Na^+^]_I_ amplitude by 27% (for $I_{max}^{pump}$= 0.3 nA, 90% CI: 1.75–2.44, range: 1.34–3.85, *n* = 13; for $I_{max}^{pump}$= 0.7 nA, 90% CI: 1.35–1.94, range: 1.02–2.82, *n* = 13; Figs. 3, 4*C*,*D*), and increased the amplitude of oscillations of I_pump_ by 27% (for $I_{max}^{pump}$= 0.3 nA, 90% CI: 0.07–0.10, range: 0.05–0.16, *n* = 13; for $I_{max}^{pump}$= 0.7 nA, 90% CI: 0.12–0.15, range: 0.08–0.19, *n* = 13; Fig. 4*F*) while not changing the median I_pump_ (Fig. 4*E*). The bursting characteristics were not normally distributed and exhibited a positive skew. To assess statistical differences between groups, we performed a nonparametric Mann–Whitney *U* test. We compared the low and high points used to compute the percent change for various parameters, such as BD, IBI, baseline [Na^+^]_i_, [Na^+^]_i_ amplitude, baseline I_pump_, and I_pump_ amplitude. Significant differences were found in their test statistic *U*-values, associated p-values, and sample sizes: BD (*U* = 156.0, *p* = 5.5 × 10^−6^, *n* = 44), IBI (*U* = 156.0, *p* = 5.5 × 10^−6^, *n* = 44), Baseline [Na^+^]_i_ (*U* = 156.0, *p* = 5.5 × 10^−6^, *n* = 44), [Na^+^]_i_ amplitude of oscillations (*U* = 156.0, *p* = 5.5 × 10^−6^, *n* = 44), Baseline I_pump_ (*U* = 78.0, *p* = 1.0, *n* = 44), I_pump_ amplitude (*U* = 13.0, *p* = 0.00015, *n* = 44).

### A simple HN neuron model explains how $I_{max}^{pump}$ controls burst duration and interburst interval

**Figure 5.**
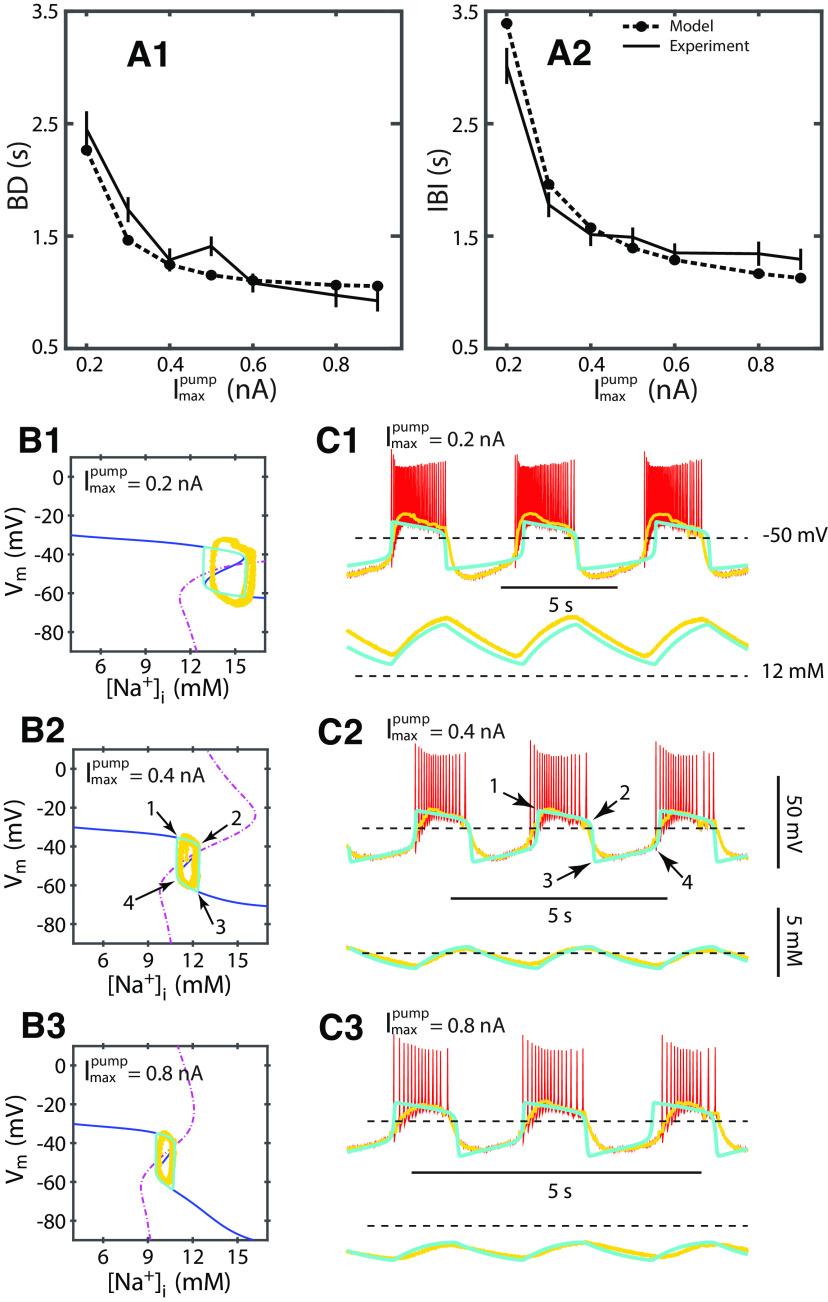
A two-dimensional HN model unravels the dynamics of the interaction of the persistent Na^+^ and pump currents in a leech heart interneuron with I_NaP_ and I_pump_ augmented by dynamic clamp. Our model explains the speeding up of the HA bursting observed in the dynamic clamp experiments with increasing $I_{max}^{pump}$. ***A***, Envelope characteristics of the 2D model (cyan) match corresponding bursting characteristics obtained from a single dynamic clamp experiment: increasing dynamic-clamp injected $I_{max}^{pump}$ decreases BD and IBI. These graphs show the effect of increasing $I_{max}^{pump}$ from 0.3 to 0.9 nA, while keeping dynamic clamp injected ${\bar{g}}_{NaP}$ at 6 nS on BD (***A1***) and IBI (***A2***). The model mimicked the parameter changes of experimental protocol without change any other model parameters. These graphs demonstrate a good fit between experiment and model (BD relative error = 0.1004, IBI relative error = 0.0913). ***B1–B3***, Phase portraits showing model periodic orbits (limit cycles) and nullclines and projected experimental data, accompanied by timeseries of the experimental and model membrane potential, membrane potential envelope, and [Na^+^]_i_ (***C1–C3***) for three different values of injected I_NaP_. ***C1–C3***, Timeseries of experimental (yellow) and simulated (cyan) V_m_ envelope for three levels of $I_{max}^{pump}$. ***B2***, ***C2***, The V-nullcline is the Z-shaped blue curve and the [Na^+^]_i_-nullcline is the magenta curve. The intersection between these nullclines determines location of the unstable steady state within the limit cycle. Their geometry defines the trajectory of the limit cycle of the model (Fig. 6*B*).

We investigated the oscillatory properties of the interaction of I_NaP_ and I_pump_ in a simple model with two dynamic variables, membrane potential, V_m_, and intracellular Na^+^ concentration, [Na^+^]_i_. We curve-fitted this 2D model to match experimental data (Materials and Methods). Our simulations emulate experiments following the protocol 2 (Materials and Methods): we systematically varied $I_{max}^{pump}$ from 0.2 to 0.9 nA but kept all other parameters constant, including conductance of injected I_NaP_, ${\bar{g}}_{NaP}$= 6 nS. The 2D model has a subset of currents (I_h_, I_NaP-native_, I_K2_, I_NaF_, I_leak-K_, and I_leak-Na_ components) incorporated as steady-state currents with their kinetics taken from the canonical model. They are incorporated to represent cell-to-cell variability in experiments. The injected persistent sodium current (I_NaP_) and the injected Na^+^/K^+^ pump current (I_pump_) had the same parameters as in experiments. The optimization algorithm simultaneously matched the waveforms of the experimental voltage envelope and [Na^+^]_i_, BD, and IBI with their simulated counterparts. Figure 5 shows an example of a model optimized to one representative experiment, including eight values of $I_{max}^{pump}$. The simulated BD and IBI follow the same trend of the dependence on $I_{max}^{pump}$ as observed experimentally and are within range of experimentally observed characteristics (relative error: BD = 0.10, IBI = 0.09; Fig. 5*A1*,*A2*).

To relate the 2D model dynamics to experimental data, we outlined its phase portrait. We considered a phase plane of the two phase variables ([Na^+^]_i_, V_m_), where each point represents a certain state of the neuron described by the two variables. We plotted its oscillatory trajectory projected along with the envelopes of experimentally obtained bursting waveforms (Fig. 5*B1–B3*,*C1–C3*). The oscillatory motion of the 2D model on the phase plane is governed by Equations 3 and 4 describing the dependence of the speed of change of the phase variables V_m_ and [Na^+^]_I_, respectively, on the position of the system on the plane. The nullclines of the system determine borders between domains on the phase plane where the corresponding variables change the sign of their speed, e.g., reverse directions of motion, since by definition a nullcline of a phase variable is a set of points on the phase plane where the derivative of the variable is zero. The intersection(s) between the two nullclines defines the stationary steady state(s) of the 2D system. The V_m_-nullcline marks the model’s states at which the inward currents are in precise balance with outward currents and, thus, the total current is zero (Eq. 3). It separates the domains with negative and positive derivative of V_m_ . The V_m_-nullcline has a z-shape, the upper and lower branches of z-shaped nullcline attract the trajectories (stable branches) since below them, the derivative of V_m_ is positive and V_m_ increases, while above them, the derivative of V_m_ is negative, and V_m_ decreases (repolarizes; Fig. 5*B2*). and with the described directions of the speed of V_m_. The middle (unstable) branch, connecting the two branches at the knee points, repels the trajectories since the directions of the membrane potential derivative are away from it. It establishes a threshold between depolarized and hyperpolarized states of the neuron or phases of oscillatory activity. Similarly, the [Na^+^]_i_-nullcline marks the neuron’s states at which the inward Na^+^ fluxes are in precise balance with outward Na^+^ fluxes and the total Na^+^ flux is zero (Eq. 4). On the left side of the [Na^+^]_i_-nullcline, the speed of [Na^+^]_i_ is positive and [Na^+^]_i_ increases, and on the right side of this nullcline, the speed reverses the sign and [Na^+^]_i_ decreases. The membrane potential V_m_ changes much faster than the intracellular Na^+^ concentration, since the factors determining time scales of the equations are more than three orders of magnitude different, $C_{m}$ ≪ vF. Thus, trajectories almost immediately reach either the upper or lower branch of the z-shaped V_m_-nullcline, reaching a state close to the balance of the inward and outward currents without notable change in the intracellular Na^+^ concentration. The phase point representing the model’s state will almost always be located in the vicinity of the stable branches (Fenichel, 1979). If the [Na^+^]_i_-nullcline intersects the V_m_-nullcline at the middle branch, the intersection point is an unstable stationary state. After reaching one of the branches of V_m_-nullcline, the trajectory will evolve so, that the [Na^+^]_i_ will slowly change according to the sign of its derivative, while staying in the close vicinity to the V_m_-nullcline. These dynamics produces a closed periodic orbit (limit cycle) on the phase plane (Fig. 5*B1–B3*) and simultaneous oscillations of V_m_ and [Na^+^]_i_ (Fig. 5*C1–C3*).

The limit cycle comprises four sections. Two of them are slow and follow the upper and lower branches of the z-shaped V_m_-nullcline: the upper slow section of the orbit represents the depolarized, spiking phase and the lower slow section represents hyperpolarized, quiescent phase of the bursting cycle. In contrast, the fast sections represent the rapid transitions between spiking and silent phases roughly at the knee points which serve as the thresholds for these transitions (Figs. 5*B2*, 6*C2*). Thus, these knee points of the V_m_-nullcline determine the range and thus amplitude of the oscillations of [Na^+^]_i_. The durations of the depolarized (BD) and hyperpolarized (IBI) phases are similarly determined by the [Na^+^]_i_ amplitude and distinctly determined by the speed of [Na^+^]_i_ along the corresponding upper and lower sections of the V_m_-nullcline. Consideration of these two controlling factors explains the dependence of the BD and IBI on $I_{max}^{pump}$.

**Figure 6.**
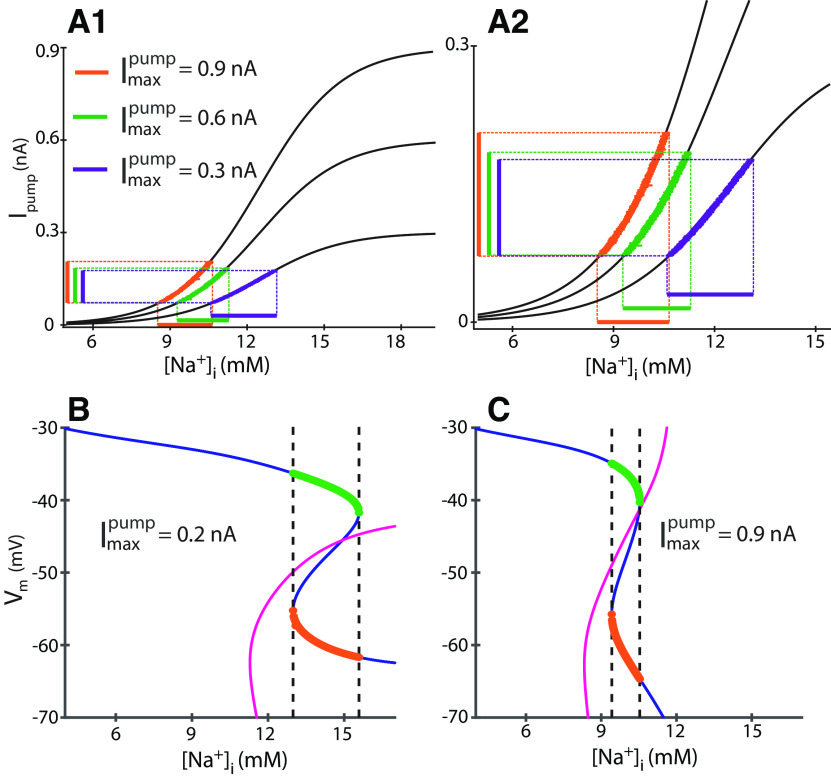
Amplitude of ${\left[Na^{+}\right]}_{i}$ in experimental data, and model nullclines in the phase plane. ***A1***, Black curves are activation functions for three levels of $I_{max}^{pump}$: 0.3, 0.6, and 0.9 nA, showing I_pump_ as a function of [Na^+^]_i_, color coded for each $I_{max}^{pump}$. Vertical projections to the left demark maximum and minimum I_pump_ values. Horizontal projections to the bottom demark maximum and minimum [Na^+^]_i_ values. ***A2***, Zoomed inset of ***A1***. These curves and ranges show the dependence of Na^+^/K^+^ pump activity as a function of [Na^+^]_i_. Consistent with Figure 5, the range of [Na^+^]_i_ at $I_{max}^{pump}$ 0.6 and 0.9 nA decreased relative to 0.3 nA, while the range of I_pump_ increased. ***B***, ***C***, Blue curve is the V-nullcline and magenta curve is the [Na^+^]_i_ nullcline. Green filled circles are 100 interpolated data points used to estimate the speed of [Na^+^]_i_, $\frac{d{\left[Na^{+}\right]}_{i}}{dt}$, during the burst duration; orange circles are the 100 data points used to estimate the rate of change of [Na^+^]_i_ during the interburst interval. Vertical dashed lines project from the nullcline knee points and delimit the maxima and minima of [Na^+^]_i_ and thus its amplitude. ***B***, $I_{max}^{pump}$: 0.2 nA. ***C***, $I_{max}^{pump}$: 0.9 nA.

We investigated how the phase portrait evolved along with the changes of $I_{max}^{pump}$ from 0.2 to 0.9 nA (Fig. 5*B1–B3*). The range of the oscillations of [Na^+^]_i_ shortens with the increase of $I_{max}^{pump}$ as the nullclines shift to the left along the [Na^+^]_i_ axis and the distance between the knee points shrinks (Fig. 5*B1–B3*; Table 6). The parameter $I_{max}^{pump}$ scales the sigmoidal activation curve of the Na^+^/K^+^ pump, so that with larger values of $I_{max}^{pump}$, the pump current reaches the same range at the smaller corresponding concentration values and more narrow range of [Na^+^]_i_ (Fig. 6*A1*,*A2*). As we increased $I_{max}^{pump}$ from 0.3 to 0.9 nA, the I_pump_ activation curves became steeper while the median [Na^+^]_i_ and [Na^+^]_i_ amplitude decreased; Figure 6*A1*,*A2* shows the activation curves for three levels of $I_{max}^{pump}$. The vertical and horizontal projections delimit the minimum and maximum I_pump_ and [Na^+^]_i_, respectively, and facilitate visual comparison between $I_{max}^{pump}$ 0.3, 0.6, and 0.9 nA. This comparison is consistent with the above analysis (Fig. 4*C–F*), where we determined that increasing $I_{max}^{pump}$ decreases the [Na^+^]_i_ median and amplitude, while the amplitude of I_pump_ increases, and the median of I_pump_ remains unchanged. Altogether these analyses demonstrate that increasing $I_{max}^{pump}$ produces a more significant I_pump_, which compresses and shifts the [Na^+^]_i_ oscillations. This scaling effect explains the shift of the knee-points of the V_m_-nullcline toward smaller intracellular Na^+^ concentration and the compression of the distance between them (Fig. 6*B*,*C*). This factor of the amplitude of [Na^+^]_i_ predicts simultaneous shortening of the BD and IBI with the same rate relative to the increase of $I_{max}^{pump}$.

**Table 6:** Relative change of the amplitude of the oscillations of [Na^+^]_i_ and the speed $\frac{d{\left[Na^{+}\right]}_{i}}{dt}$**, during the burst and during interburst interval along with the increase of** $I_{max}^{pump}$

$I_{max}^{pump}$ (nA)	δ [Na^+^]_i_amplitude	$\text{δ}\frac{d{\left[Na^{+}\right]}_{i}}{dt}$ Burst	$\text{δ}\frac{d{\left[Na^{+}\right]}_{i}}{dt}$ IB-Interval
0.2	1	1	1
0.3	0.63456	1.0455	1.1615
0.4	0.53326	1.068	1.2439
0.5	0.4881	1.082	1.3013
0.6	0.46322	1.0918	1.3458
0.8	0.43753	1.1053	1.414
0.9	0.43034	1.1104	1.4415

The second controlling factor describes how the speed, $\frac{d{\left[Na^{+}\right]}_{i}}{dt}$, along the slow sections of the limit cycle changes with the increase of $I_{max}^{pump}$. These changes are caused by the change of the magnitude of the total Na^+^ flux (Eq. 4) and can be different for the depolarized and hyperpolarized phases of the cycle. More depolarized membrane potential produces higher Na^+^ influx through the fast Na^+^ and persistent Na^+^ currents and supports the motion along the cycle. With the increased $I_{max}^{pump},\;$ the obtained Na^+^ concentration activates stronger pump current creating stronger Na^+^ efflux producing slowing down (opposing) effect on the motion during depolarized phase and speeding up (supporting) effect on the motion during hyperpolarized phase of the cycle. Notably, at the hyperpolarized membrane potentials the fast Na^+^ current is fully deactivated and the persistent Na^+^ current is partially deactivated. Along with the above described compression (Fig. 6*B*,*C*) the hyperpolarized section of the cycle expands toward a more hyperpolarized membrane potential where the persistent Na^+^ current is further diminished by deactivation, produces smaller Na^+^ influx and thus suggests a steeper dependence of the change of speed, $\frac{d{\left[Na^{+}\right]}_{i}}{dt}$, on $I_{max}^{pump}$ over the hyperpolarized section (Fig. 6*C*).

We evaluated relative contributions of this second factor on BDs and IBIs by considering the slow motion of the phase point between the knee points along with the depolarized and hyperpolarized branches of the V_m_-nullcline, respectively, with the speed $\frac{d{\left[Na^{+}\right]}_{i}}{dt}$. The speed changes along the sections of the branches and, for our approximations, we evaluated it at 200 points (100 points per branch section) distributed with equal intervals in terms of ${\left[Na^{+}\right]}_{i}\;$ between the knee points (vertical dashed lines), using Equation 4 (Fig. 6*B*,*C*). The computed average speeds for these two sections over these points show that with the increase of $I_{max}^{pump}$, the average speed over the hyperpolarized section increased with a higher rate than over the depolarized one (Table 6). This factor predicts that IBI shortens faster than BD relative to the increase of $I_{max}^{pump}$.

**Figure 7.**
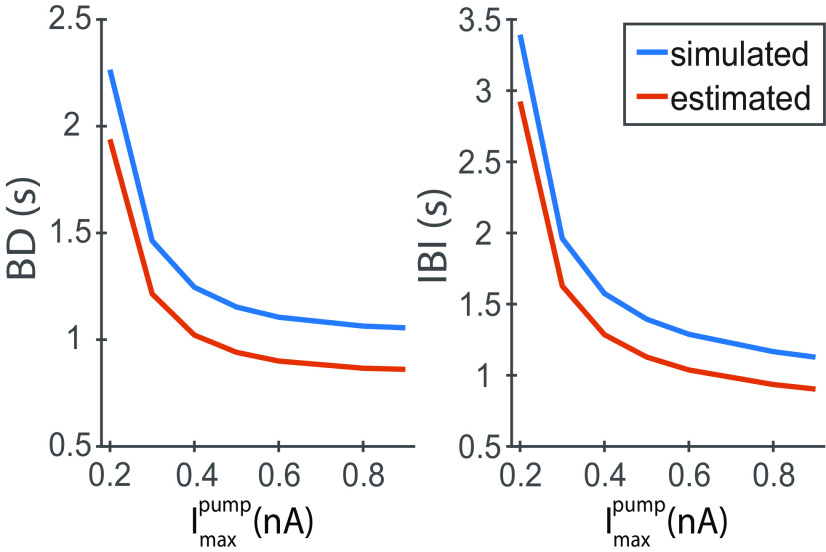
The estimated BD and IBI preserve the dependence on $I_{max}^{pump}$ observed experimentally and confirmed by the 2D HN model. The estimated BD and IBI from nullcline analysis preserve the trend observed on the BD and IBI from simulations of the 2D model. Importantly, these estimates were computed by interpolating 100 points from the nullclines’ geometry and demonstrating that the [Na^+^]_i_ speed plays an essential role in determining the BD and IBI. To compute these estimates, we used the points illustrated in Figure 6 (V and [Na^+^]_i_ coordinates) to compute the currents and [Na^+^]_i_ speed. We divided the change in [Na^+^]_i_ amplitude by the [Na^+^]_i_ speed to get the estimated BD and IBI.

**Figure 8.**
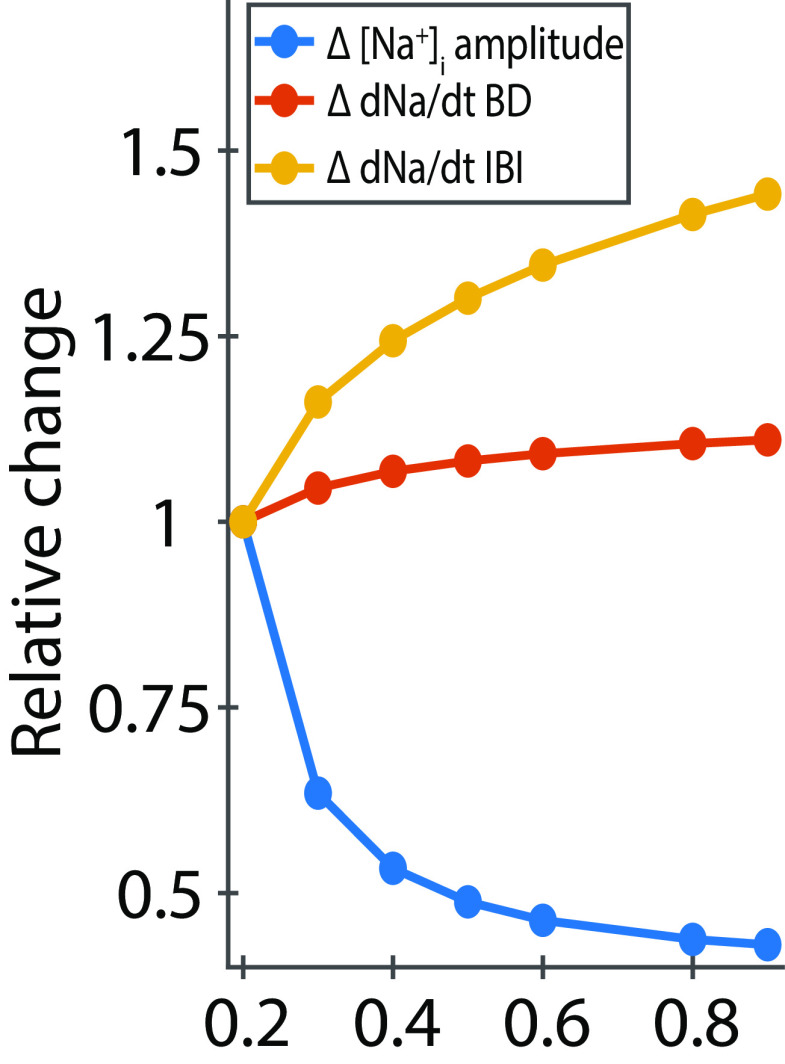
[Na^+^]_i_ amplitude decreases 57%, the [Na^+^]_i_ speed $\left(\frac{dNa}{dt}\right)$ during the burst increases 11%, and the [Na^+^]_i_ speed during the interburst interval increases significantly 44% as a function of $I_{max}^{pump}$. These curves illustrate the relative change of the [Na^+^]_i_ amplitude and [Na^+^]_i_ speed as a function of $I_{max}^{pump}$. We collected the [Na^+^]_i_ amplitude and speed during the burst and interburst interval computed from the 100 points as depicted in Figure 6*B* and *C*. To compute the relative change, we divided each [Na^+^]_i_ amplitude or speed by its correspondent collected at the initial point ( $I_{max}^{pump}=$ 0.2 nA).

Approximation of the BD and IBI by the integration of the slow motion along with the slow branches of the V_m_-nullcline roughly matches the values obtained from the simulated cycle with the relative errors of 18% (Table 7; Figs. 7, 8). With $I_{max}^{pump}$ growing from 0.2 to 0.9 nA, the amplitude of oscillations of [Na^+^]_i_ shrinks by 57%, the average speed, $\frac{d{\left[Na^{+}\right]}_{i}}{dt}$, during the burst increases by roughly 11%, whereas during the interburst interval, it increases by 56% (Table 6; Fig. 7). Altogether, the two factors, the dependence of the intracellular Na^+^ concentration amplitude and speed on $I_{max}^{pump}$, explain the mechanism underlying the decrease of BD and IBI with the increase of $I_{max}^{pump}$ in the dynamic-clamp experiments. The amplitude of [Na^+^]_i_ is the dominating factor determining the burst duration, while both the [Na^+^]_i_ amplitude and speed determine the interburst interval.

**Table 7:** Simulated and approximated characteristics of bursting (BD and IBI)

$I_{max}^{pump}$ (nA)	Simulated BD (s)	Approximated BD (s)	Simulated IBI (s)	Approximated IBI (s)
0.2	2.26	1.94	3.39	2.92
0.3	1.46	1.21	1.96	1.63
0.4	1.25	1.02	1.57	1.28
0.5	1.15	0.94	1.39	1.13
0.6	1.11	0.90	1.29	1.04
0.8	1.06	0.87	1.17	0.93
0.9	1.06	0.86	1.13	0.90

The estimations were done based on measurements made at 100 points on the depolarized and hyperpolarized branches of V_m_-nullcline between the knee-points. The average relative error between the simulated and approximate measurements were 18%.

## Discussion

All rhythmically active neuronal networks, like the CPG studied here, require robust neuronal mechanisms for burst formation and termination. Associated with bursts are changes in intracellular Na^+^ concentration so that Na^+^ influxes and effluxes, while oscillatory, are balanced on the cycle-to-cycle basis. Na^+^ efflux is mostly taken care of by the Na^+^/K^+^ pump, which is a basic cellular engine maintaining the physiological gradients of Na^+^ and K^+^ ions across the membrane. Because of unbalanced exchange of 3 Na^+^ ions moved out for two K^+^ ions returned back into the cell, the pump generates an outward current whenever it operates and because it responds by increasing its activity whenever there is Na^+^ buildup in the cell it responds dynamically to spiking and bursting activity. Recent studies show that the Na^+^/K^+^ pump current contributes to the dynamics of neurons and is a target of neuromodulation (Bertorello and Aperia, 1990; Bertorello et al., 1990; Catarsi and Brunelli, 1991; Tobin and Calabrese, 2005; Arganda et al., 2007; Scuri et al., 2007; Hazelwood et al., 2008; Pulver and Griffith, 2010; Forrest et al., 2012; L.N. Zhang et al., 2012, 2013; H.Y. Zhang et al., 2015; Picton et al., 2017a,b; Sharples et al., 2021; Hachoumi et al., 2022). For example, the pump has been shown to play an important role in sensory information coding (Arganda et al., 2007; Scuri et al., 2007), and the dynamics of short-term memory in a rhythmic motor activity (Pulver and Griffith, 2010; H.Y. Zhang and Sillar, 2012; Picton et al., 2017a; H.Y. Zhang et al., 2017; Hachoumi et al., 2022). The Na^+^/K^+^ pump current plays an atypical role among membrane currents because it is continuously active across the entire voltage range of neuronal activity, while other currents have distinctive voltage ranges of operation and may inactivate. Moreover, because the pump is activity dependent, adjusting to [Na^+^]_i_ that builds through spiking and bursting activity, it dynamically adjusts to that activity. Thus, in bursting neurons the pump can potentially contribute to cessation of the burst phase and the prolongation of the interburst interval and must be considered as part of the burst mechanism on the level of a single cell dynamics (Li et al., 1996; Fröhlich et al., 2006; Barreto and Cressman, 2011; Krishnan and Bazhenov, 2011; Yu et al., 2012; Jasinski et al., 2013; Sharples et al., 2021). Here, using bursting HN interneurons from the leech heartbeat CPG in conjunction with intracellular electrophysiology, dynamic clamp, and modeling, we identify a key interaction with persistent Na^+^ current, a current which supports spiking and burst formation and is also active over a very broad voltage range and thus contributes directly to [Na^+^]_i_ even below spiking threshold. A quantitative analysis of this interaction was possible with our recently developed dynamic clamp implementation estimating Na^+^ fluxes and [Na^+^]_i_ and injecting simulated pump and persistent Na^+^ currents into the cell in real time.

Leech HN interneurons are organized into a functional CPG by strong inhibitory synaptic connections, but each HN interneuron is capable of producing rhythmic bursting autonomously when synaptically isolated with bicuculline (Cymbalyuk et al., 2002) or by ganglionic isolation as in the HN(7) interneurons studied here. The dynamics of the HN neurons are well described; the ionic currents and their inhibitory synaptic interactions have been characterized using voltage clamp and these data have been used to build experimentally useful biophysical models of an HN neuron and an HN mutually inhibitory pair or half-center oscillator (HCO; A.A. Hill et al., 2001). This model has demonstrated predictive power for new experiments unveiling roles of interaction of specific ionic currents in control of bursting properties of HN and HN-HCO (A.A. Hill et al., 2001; Cymbalyuk et al., 2002; Simoni et al., 2004; Sorensen et al., 2004; Olypher et al., 2006; Kueh et al., 2016; Ellingson et al., 2021). In HN interneurons of the leech heartbeat CPG, persistent Na^+^ and Na^+^/K^+^ pump currents (I_NaP_ and I_pump_) are native currents that are targets of neuromodulation by endogenous neuropeptides in the leech (Opdyke and Calabrese, 1994; Schmidt et al., 1995; Nadim and Calabrese, 1997; Tobin and Calabrese, 2005; Kueh et al., 2016; Ellingson et al., 2021). By applying the dynamic clamp technique, we showed that their interaction undergirds bursting activity. While allowing manipulation of neuronal activity, invasive recording tools, such as sharp intracellular electrodes, can unintentionally affect measured neuronal activity (Cymbalyuk et al., 2002; Pusuluri et al., 2021). Here, we demonstrated that appropriate dynamic clamp implementation of these inward and outward currents (I_NaP_ and I_pump_) can alleviate the sharp electrode membrane damage and restore typical bursting of HN neurons (Low-Voltage Amplitude bursting regime) in which neuronal excitability and ability to repolarize are both compromised by the nonspecific leak current with the reversal potential 0 mV introduced by sharp electrode penetration (Cymbalyuk et al., 2002; Fig. 1*A*). I_pump_ responds dynamically to [Na^+^]_i_, differentiating it from a steady (or voltage-gated) outward current. Thus, pump activity (as embodied in the parameter $I_{max}^{pump}$) modulates excitability in HN neurons; increasing pump activity diminishes excitability while decreasing the pump activity enhances excitability. Our experiments demonstrated that excitability must be preserved by mutually counterbalancing currents if bursting is to be maintained with sharp electrode recordings. This finding suggests that this interaction creates a mechanism which flexibly support bursting activity across a wide range of dynamical parameters. This result also accentuates that the dynamic clamp technique is uniquely valuable for prototyping and testing brain-machine interface techniques (Prinz et al., 2004a; Erazo-Toscano et al., 2021).

Sufficient increase of I_NaP_ and I_pump_ with dynamic clamp causes transition of HN neurons into an intense bursting regime. In every HN neuron that we studied, we found that joint upregulation of I_NaP_ and I_pump_, specifically by increasing $I_{max}^{pump}$ to at least 0.1 nA and ${\bar{g}}_{NaP}$ >5.0 nS, gave rise to this new bursting regime, the high voltage amplitude (HA) bursting (Figs. 1*C*, 2). In addition, we showed that once in the HA bursting regime, increasing $I_{max}^{pump}$ decreases the burst duration (BD) and the interburst interval (IBI), effectively speeding up the rhythm of the oscillation; this mechanism allows robust and flexible control of the pace of the neuronal bursting (Figs. 4, 5).

Depending on environmental factors or behavioral goals, the leech heartbeat CPG should be able to adjust the pacing of the heartbeat. Our experiments demonstrate that HN neurons are capable of two bursting regimes differentiated by their level of excitability signified by the intraburst spike frequency. In the LA, excitability is low, and bursts are long with low spike frequency. In contrast, in the HA, excitability is high, bursts are short with high spike frequency. Because HN neurons project several segments/ganglia to engage other HN neurons of the CPG and motor neurons through inhibitory synapses and electrical junctions, spikes are the major mode of communication in the network, thus the differences in spike frequency between the LA and HA bursting regimes suggest these bursting regimes could support different functions.

### The Na^+^/K^+^ pump monitors cellular activity and provides a negative feedback

The Na^+^/K^+^ pump is generally recognized as playing a critical role in self-regulating neuronal activity (Skou, 1988; Brodsky and Guidotti, 1990; Luger, 1991; Glynn, 1993), but less often is the underlying [Na^+^]_i_ that it regulates considered as a measure of neuronal activity. By generating outward current in response to raised spiking activity, the Na^+^/K^+^ pump produces negative feedback which could stabilize functional regimes. Malfunctions in dynamics of [Na^+^]_i_ can lead to dynamical diseases (Belair et al., 1995). For example, functional regimes can make transitions into seizure regimes or coexist with them (Hahn and Durand, 2001; Forger and Paydarfar, 2004; Fröhlich and Bazhenov, 2006; Ziburkus et al., 2006; Cressman et al., 2009; Ullah et al., 2009; Fröhlich et al., 2010; Krishnan and Bazhenov, 2011). During seizures the Na^+^/K^+^ pump can play a crucial role in termination and in postictal depression (Cressman et al., 2009; Ullah et al., 2009; Fröhlich et al., 2010; Krishnan and Bazhenov, 2011; Krishnan et al., 2015). Based on our results we hypothesize that when neuromodulation targets Na^+^/K^+^ pump activity, co-modulation of additional current(s) could mitigate dangerous consequences for the dynamics of the functional regime. For example, in the leech heartbeat CPG, myomodulin, an endogenous leech neuropeptide, reduces the pump’s activity and enhances I_h_; in synaptically isolated HN neurons, weak bursting rhythms become robust with myomodulin application (Tobin and Calabrese, 2005). The net effect is that myomodulin supports functional bursting, speeds up bursting pattern, and avoids dysfunctional seizure-like and asymmetrical bursting regimes in HN neurons (Tobin and Calabrese, 2005; Kueh et al., 2016; Ellingson et al., 2021). In contrast, enhancing only one current can disrupt functional activity in neurons. The predicted new HA bursting might be invoked in the cases of high neuromodulation where the Na^+^-carrying currents are activated, and pump current is reduced or is challenged with Na^+^ influx. This regime has not been demonstrated yet in experiments with the leech heartbeat HN neurons, and further investigation is needed to explore the full range of conditions that can lead to the generation of HA bursting activity in these neurons.

The importance of [Na^+^]_i_ as the measure of spiking activity in production of the negative feedback is also evident from the studies of the Na^+^-dependent K^+^ (I_KNa_) currents which read out this proxy and synergize with the pump as suggested by Hage and Salkoff (2012). In the leech heartbeat neuronal model, a similar interaction is reported with the noninactivating K^+^ (I_K2_) current providing negative feedback based on integration of membrane potential during spiking. This mechanism also requires coordination of conductance values of the persistent Na^+^ and K^+^ currents and the leak current (Doloc-Mihu and Calabrese, 2014, 2016). Further, [Na^+^]_i_ activates the Na^+^/K^+^ pump, and because the pump activity consumes ATP and oxygen, [Na^+^]_i_ may act as an yet undescribed homeostatic mechanism. The impact of these [Na^+^]_i_ fluctuations on cellular activity remains unexplored mainly, but our work suggests that it may be a fruitful avenue of future research.

Our work estimated underlying oscillations of [Na^+^]_i_ that accompany bursting cycles. The amplitude of these rhythmic fluctuations is determined by burst intensity (burst duration, voltage envelope amplitude, and spike frequency; Fig. 4) and thus serve as a proxy for the intensity of the bursting regime. The dynamics of the intracellular Na^+^ concentration along with the membrane potential explains the properties of the HA bursting. To describe the core of this mechanisms, we assembled a biophysical model with just two state variables (2D model), i.e., the membrane potential and the intracellular Na^+^ concentration ([Na^+^]_i_; Fig. 5). This allowed us to apply phase plane analysis and to investigate self-sustained oscillations. The 2D model captured and explained the changes in each of the two burst characteristics (burst duration, and interburst interval), and two waveform characteristics (voltage envelope and intracellular sodium oscillation amplitudes) within the dynamic-clamp experiments injecting the Na^+^/K^+^ pump current scaled by $I_{max}^{pump}$. The 2D model quantitively reproduced the dependence of the burst characteristics on $I_{max}^{pump}$ measured experimentally. Our model shows that one can estimate the burst duration and interburst interval based on the geometry of the nullclines. These results suggest that during and between the bursts the inward and outward currents are almost in perfect balance and depolarization and repolarization is driven by the relatively slow change in the intracellular Na^+^ concentration according to fast dynamics of the membrane potential and the slow dynamics of Na^+^ concentration and is well described as dynamics of a relaxation oscillator (Bogoliubov and Mitropolsky, 1961; Rinzel, 1986). This analysis emphasizes that the range of the intracellular Na^+^ concentration as the major factor responsible for the simultaneous shortening of the burst duration and interburst interval of HA bursting with the up-scaling of Na^+^/K^+^ pump activity, and the disbalance of the Na^+^ influx and efflux as the second factor controlling the speed of concentration change during and between the bursts. In HA bursting, I_NaP_ and I_pump_ are near balance during the burst and the interburst interval, driving burst initiation and burst termination. The conventional understanding of control over bursting characteristics by conductance-based currents is fundamentally different; for example, activation of outward I_K_ or inactivation of inward I_Ca_ may terminate a burst, determined by activation/inactivation variables that change over time with a time constant (A.A. Hill et al., 2001; Olypher et al., 2006). In contrast, in the mechanism formed by the interaction of I_NaP_ and I_pump_, the two currents are balanced, supporting the burst, and the burst terminates through the saddle-node bifurcation in the slow subsystem. Considering Na^+^ concentration allowed us to thoroughly characterize the dynamics governing burst characteristics in HN neurons, and with this we predict transition of these neurons into a high excitability bursting regime under the dynamical challenge posed by neuromodulation. We propose that there are at least two different bursting patterns, and that the roles of ionic currents changes with the switch between LA and HA potentially by neuromodulation. We dissected this new HA bursting mechanism down to interaction I_NaP_ and I_pump_ through intracellular Na^+^ concentration which allowed us to quantitatively match temporal properties like burst duration and interburst interval along with the change of the maximal pump current. Since this description quantitatively describes changes of the burst duration, we conclude that this interaction controls the termination of the burst and interburst interval. Since, this mechanism produces shorter bursts and interburst intervals, it overrides the mechanisms described for LA bursting (A.A. Hill et al., 2001).

## Synthesis

Reviewing Editor: Silvia Pagliardini, University of Alberta Decisions are customarily a result of the Reviewing Editor and the peer reviewers coming together and discussing their recommendations until a consensus is reached. When revisions are invited, a fact-based synthesis statement explaining their decision and outlining what is needed to prepare a revision will be listed below. The following reviewer(s) agreed to reveal their identity: Muriel Thoby-Brisson, Anna Schneider.

In the paper entitled « Robust bursting dynamics based on the interaction of persistent Na ans Na/K pump currents: a dynamic clamp approach”, the authors used bursting HN interneurons from the leech heartbeat CPG in conjunction with intracellular electrophysiology, dynamic clamp, and modeling. They identified a key interplay between the persistent sodium current Ip and INa/Kpump (an outward current activated by the intracellular sodium concentration) to support functional bursting. They show that a combinatorial increase in Ip and Ipump in a living synaptically isolated HN neuron induces the transition towards a new bursting regime characterized by a high spike frequency and a large amplitude of the membrane potential oscillations. With a computational model they explain the potential underlying mechanisms to control bursts parameters. This would be an efficient mechanism to provide a higher flexibility to CPGs involved in generating rhythmically organized activities, such as breathing or heart-beating.

While authors present a thorough experimental design the presentation of results lacks statistics and sufficient methodological explanations, and quantifications are incomplete.

Major:

- Statistical tests are missing:

o p16: Are LVA and HVA states statistically different to support the authors’ claim that these are two different bursting regimes? The authors provide means and SD but no statistics or sample size.

o p16: “the scatterplots [...] indicate the differences”. Do the statistics on the data in Fig 2B and C to demonstrate that the differences are significant.

o p17f: do the statistics for the percent changes.

- Quantifications are missing or incomplete for some of the claims the authors make:

o p15: Coefficient of variation. What is the CV of the parameter in control, what threshold for CV is used to make the claim that something is regular or irregular or that CV is high? Since CV can theoretically range from 0 to infinity a CV of 0.38 has no meaning without comparing it to the CV of what the authors’ claim to be regular activity.

o p16: Same comment as above for the variability of cycle period.

o p17f: Give confidence intervals and n for the percentage change.

o p23: Table 5 caption: Give confidence interval for the 18% relative error.

- Methods are insufficiently explained:

o p7: data analysis: Please state how many cycles or seconds were analyzed per animal, the criteria to remove datasets (e.g. those indicated by crosses in Fig 2D) and what exactly the dots in the scatterplots represent. This is necessary information to judge the quality of the presented results.

- Terminology: in a vast majority of publications on membrane conductances and bursting activities the persistent sodium current is referred as INap. Here it is called Ip. It would be judicious to use the same terminology as commonly used.

In the same vain, the authors named their two main bursting regimes as LVA and HVA for Low Voltage Amplitude and High Voltage Amplitude, respectively. Here again those acronyms are currently used for low and high voltage activated calcium current. To avoid any confusion would it be possible for the authors to propose another name.

- page 16: The authors are able to reinstate bursting activity in HN neurons when recorded with a sharp electrode, an approach known to prevent bursting activity in these neurons and to induce a tonic discharge instead. They do this by introducing relatively small INap and Ipump current with dynamic clamp. What is the relevance of doing so? What are the interpretation of the authors to explain this effect? What is the functional link between INap+Ipump vs the shunting leak current supposedly activated by the recording sharp micropipette?

-Fig 1: the authors described the emergence of a new bursting regime with a higher spike frequency and a higher amplitude of the envelope underlying the oscillations. Does this specific discharge pattern exist in “real” HN interneurons? Does it correspond to any physiological activity? Can this pattern be expressed under a specific neuromodulatory environment? What would be the physiological role of this HVA bursting activity? The authors must at least discuss these points and should make an effort to associate this bursting activity with any functional role in the animal.

- page 26: the authors propose that in bursting neurons the pump can potentially contribute to cessation of the burst phase and prolongation of the interburst interval and must be considered as part of the endogenous bursting mechanism. Then how does this interfere with the other conductances involved in burst termination (such as the different potassium associated conductances)? How can the authors be sure that during HVA and LVA bursting activities completely distinct sets of conductances can produce either high amplitude high frequency bursts vs low amplitude low frequency ones, especially when membrane conductances involved are voltage-dependent?

Minor:

- Please include line numbers

- p5: please add the amplifier’s switching frequency in DCC.

- p6: I am a little lost here, please clarify how the native currents are calculated. Are they estimated based on Hodgkin-Huxley equations and if so, which parameters were used? I don’t see any parameters or equations for the native currents in the dynamic clamp model in the supplementary data (p51ff), except I_NaF. And please state explicitly that the native currents are just estimates if that is the case.

- p7: Please add the duration of the dynamic clamp. How was steady-state determined after starting dynamic clamp or changing dynamic clamp parameters?

- page 8, first para, last sentence: “The” is repeated twice.

- p8: “a two-dimensional model”. Please state here what the two dimensions are. It becomes clear later in the manuscript but please add it here.

- p8: “we gathered the following characteristics”. Cycle period is missing in this list.

- p8: same paragraph: Please clarify how the data was normalized: Within each individual, or was each individual normalized to the mean of the population, etc.

- p8: Machine learning algorithms: Please provide the software.

- p8: How did the authors evaluate the ML algorithm’s performance? Figure 2B clearly shows that the experimental design is unbalanced which can potentially lead to overclassification in a certain direction. If they did not evaluate the performance this point should go in the Discussion.

- p9: linear regression: Please provide the software.

- p10: Equation 3: What is the division between Na and K components of leak?

- p10ff: Please swap tables 2 and 3 so that they appear in the correct order in the text.

- p12: Why was -45mV chosen as threshold?

- p14: I am confused by the n as to how many animals were used for which protocol. In the methods section, the authors state that data was normalized to Ipump_max = 0.3nA, g_P = 6nS. Here, there is only an n = 3 for the constant Ipump_max = 0.3nA and an n = 18 for g_P = 6nS. So how many animals were there in total, were they used for either constant I_pump or constant g_P or were both done in each animal?

- page 15: rewrite the sentence: “The code can will be......”

- p15: “spike frequency similar to that obtained in extracellular recordings”. This statement does not match the data from the presented experiments and the literature on p16 where LVA spike frequency = 4.1 Hz, HVA spike frequency = 17.11 Hz and Cymbalyuk et al. (2002) spike frequency = 11.4 Hz. At least for spike frq it looks like those given in the literature are right between LVA and HVA.

- p15: “dynamic clamp currents [...] support rhythmic bursting [...] undeterred by the nonspecific leak”. This statement cannot be made at this point in the manuscript since the data about the impact of I_P and I_pump is presented later. So far, the authors have only shown that I_P and I_pump injection counteract the shunting leak.

- p15: “at a constant value(Figure 1 A-C)”. Contrary to the text, panel C has a higher Ipump_max than panels A and B.

-page 17, line 13: are you sure that the reference to Figure 1B is appropriate? Shouldn’t it rather be Fig1A and C?

- p17: “to terminate bursts”. The authors have not shown at this point in the manuscript that the pump can terminate the bursts, only that there is a correlation between I_pump levels and burst termination, as they say in the same sentence. Please rephrase. The evidence comes later, when they manipulated I_pump and analyzed the model phase plane.

- p18: “kept all parameters constant”. Add “other” before parameters.

- p18: How can there be cell-to-cell variability if all parameters are constant?

- p24: “taken care by”. Add “of”.

- p27: “Because neurons communicate through spikes”. I have two comments about this section 1) Please differentiate here. Neurons also communicate through graded potentials (e.g. see Rosenbaum & Marder 2018 (https://doi.org/10.1523/JNEUROSCI.2632-17.2018) for a recent example), so the changes in the underlying voltage waveform could already be sufficient to support different functions. 2) Was HVA bursting ever observed under physiological conditions or is this some highly artificial state in which the neuron is driven by dynamic clamp?

- p51, 52: What is denoted by f_infinity in the equations of m and h gates? I assume it is a logistic function but what does it look like exactly and which of the input parameters in the parentheses is the half-activation voltage and which is steepness?

- Figure 1:

o Avoid unnecessary color coding, e.g. the bars for T, BD, IBI, the mid-burst diamonds and the spike frq traces should all be black.

- Remove the CV in panel B because this can lead to some confusion with the CVs for cycle period that are in the figure legend. Make it consistent with panels A and C and simply state that this is irregular LVA.

- The CVs for cycle period are nowhere mentioned in the text. Why are they there, what are they supposed to tell me?

-add LVA and HVA above traces in panels D1 and D2, respectively.

- Figure 2:

- What do the individual dots in panels B and C represent? Are these the averages for each animal of x cycles, or just for one cycle? Does each dot come from one combination of I_P and I_pump?

- Figure 3:

- Avoid unnecessary color coding and using the same colors for different things: In the previous figures, red was for HVA and blue for LVA. Here, it is for Vm and I_P. The color does not add any information. Either make all traces black or color the Vm trace according to LVA or HVA.

- Figure 4:

- Colors are completely unnecessary here since every individual shows the same trend and the colors are so similar that it is difficult to follow a single experiment. Show only the mean line with shaded confidence intervals.

- Swap panels E and F so they appear in the same order as in the text.

- Figure 5:

- If you use colors, use a consistent color code. Previously, cyan was used for the voltage envelope of HVA bursting. Here, it is yellow; and cyan is used for the model data.

- Supplementary Figures 1 and 2

- Please use colors that are different from

## Author Response

Dear Editors,

We would like to express our sincere appreciation to the reviewers for their time and valuable comments on the manuscript. The feedback provided helped us to improve the quality of the manuscript, and we are grateful for their input. Below, we marked copied critical parts of the comments in blue and used black and red for our responses and edits, respectively.

“While authors present a thorough experimental design the presentation of results lacks statistics and sufficient methodological explanations, and quantifications are incomplete.”

We took seriously your comment and revised the manuscript accordingly as detailed below.

Major:

- Statistical tests are missing:

o p16: Are LVA and HVA states statistically different to support the authors’ claim that these are two different bursting regimes? The authors provide means and SD but no statistics or sample size.

We used a different approach and, therefore, did not perform hypothesis testing statistics and did not evaluate the significance p-value. Instead, we used a machine learning classification algorithm, which has a report table that shows the accuracy of the classification in terms of precision, recall, F1 score, and support. We edited the ms to explain the outcome of the machine learning algorithm, starting at the line 408:

“We used a two-threshold algorithm to classify our data and train a Gaussian Naïve-Bayes supervised machine learning classification algorithm (ML, Methods). The quality of the classification is evaluated by a report table showing the accuracy of the classification in terms of precision and recall (Table 5). Precision is the ratio of true positives to the sum of true and false positives. The closer the precision score is to 1.0, the better the expected model performance. Recall is the ratio of true positives to the sum of true positives and false negatives. The closer the recall score is to 1.0, the better the expected model performance. The F1 score is the weighted harmonic mean of precision and recall. The closer the value of the F1 score is to 1.0, the better the expected performance of the model. Support is the number of actual occurrences of the class in the dataset, it diagnoses the performance evaluation process.

Table 5. Machine learning classification report

Precision Recall F1-score Support

LA 1 0.92 0.96 12

HA 0.99 1 0.99 91

Accuracy 0.99 103

macro avg 0.99 0.96 0.98 103

weighted avg 0.99 0.99 0.99 103

Looking at the precision and recall scores, one can see that for both classes (LA and HA), the precision score is above 0.9 and the recall score is above 0.9, which indicates that the model is accurately identifying both classes with high precision and recall. The F1-score, which is a weighted average of precision and recall, is also very high for both classes, indicating a high level of overall accuracy. In addition, the support value for each class is relatively high, indicating that the model was tested on a sufficient amount of data to evaluate its performance accurately. Overall, the weighted average F1-score of 0.99 indicates that the classification model is performing at a very high level of accuracy, and the accuracy of 0.99 further supports this conclusion.”

o p16: “the scatterplots [...] indicate the differences”. Do the statistics on the data in Fig 2B and C to demonstrate that the differences are significant.

To address this request, we performed a one-tailed independent samples t-test and the results are described in the ms starting from the line 429:

“To test the accuracy of the ML classification algorithm and evaluate the meaningfulness of the separation between the LA and HA groups, we performed a one-tailed independent samples t-test. The results showed a highly significant difference between the LA and HA groups (t=-13.38, p<.001, n=44), indicating that the algorithm was successful in accurately distinguishing between these two bursting patterns.”

Also, we clarified the software used in methods starting at line 257:

“All statistical analyses, including linear regression and non-parametric Mann Whitney U test were performed using Python 3.6.8 and Scipy 1.8 stats from the Anaconda distribution (anaconda.com).”

p17f: do the statistics for the percent changes.

We addressed this request in the text starting at line 465:

“The bursting characteristics were not normally distributed and exhibited a positive skew. To assess statistical differences between groups, we performed a non-parametric Mann Whitney U test. We compared the low and high points used to compute the percent change for various parameters, such as BD, IBI, baseline [Na+]i, [Na+]i amplitude, baseline Ipump, and Ipump amplitude. Significant differences were found in their test statistic U-values, associated p-values, and sample sizes: BD (U= 156.0, p= 5.5*10-6, n=44), IBI (U= 156.0, p= 5.5*10-6, n=44), Baseline [Na+]i (U= 156.0, p= 5.5*10-6, n=44), [Na+]I amplitude of oscillations (U= 156.0, p= 5.5*10-6, n=44), Baseline Ipump (U= 78.0, p= 1.0, n=44), Ipump amplitude (U= 13.0, p= 0.00015, n=44).”

- Quantifications are missing or incomplete for some of the claims the authors make:

o p15: Coefficient of variation. What is the CV of the parameter in control, what threshold for CV is used to make the claim that something is regular or irregular or that CV is high? Since CV can theoretically range from 0 to infinity a CV of 0.38 has no meaning without comparing it to the CV of what the authors’ claim to be regular activity.

o p16: Same comment as above for the variability of cycle period.

We addressed this concern with the following section placed in lines starting at line 390:

“We discarded such activities along with any other activities with high variability of the cycle period (coefficient of variation {greater than or equal to}0.25) from further analysis. The decision to use this cutoff value was informed by the comparison to the whole population, which showed a low degree of variability in the burst period. The coefficient of variation (CV) for the cycle period ranged from 0.01 to 0.43, with a median of 0.06 and 1st and 3rd quantiles of 0.04 and 0.10, respectively. These statistics indicate that the majority of the population had a relatively low degree of variation. The burst duration CV showed a wider range of variation, spanning from 0.02 to 0.95, with a median of 0.11 and 1st and 3rd quantiles of 0.07 and 0.17, respectively. Similarly, the interburst interval CV ranged from 0.02 to 0.79, with a median of 0.08 and 1st and 3rd quantiles of 0.06 and 0.17.”

o p17f: Give confidence intervals and n for the percentage change.

Please, find this information in lines 458:

“Namely, an increase of I_max^pump from 0.3 nA to 0.7 nA decreased the median [Na+]i by 18% (for I_max^pump=0.3 nA, 90 percent confidence interval (CI): 12.59 13.35, range: 11.69 14.37, n=13; for I_max^pump=0.7 nA, 90 percent CI: 10.32 10.86 , range: 9.86 11.38, n=13) and the [Na+]I amplitude by 27% (for I_max^pump=0.3 nA, 90 percent CI: 1.75 2.44, range: 1.34 3.85, n=13; for I_max^pump=0.7 nA, 90 percent CI: 1.35 1.94 , range: 1.02 2.82, n=13) (Figures 3, 4C, and 4D), and increased the amplitude of oscillations of Ipump by 27% (for I_max^pump=0.3 nA, 90 percent CI: 0.07 0.10 , range: 0.05 0.16, n=13; for I_max^pump=0.7 nA, 90 percent CI: 0.12 0.15 , range: 0.08 0.19, n=13) (Figure 4F) while not changing the median Ipump (Figure 4E).”

o p23: Table 5 caption: Give confidence interval for the 18% relative error.

The table shows the simulated and approximated characteristics of bursting (BD and IBI) for a deterministic system. The estimations were based on measurements made at 100 points on the depolarized and hyperpolarized branches of the Vm-nullcline between the knee-points. To evaluate the accuracy of the mechanism, we compared the actual model to an approximation based on the speed of change of a variable computed on the null cline. The average relative error between the simulated and approximate measurements was 18%.

Note that a statistical confidence interval for the 18% relative error is not applicable in this case, as it is based on a comparison between the actual deterministic model and an approximation.

- Methods are insufficiently explained:

o p7: data analysis: Please state how many cycles or seconds were analyzed per animal, the criteria to remove datasets (e.g. those indicated by crosses in Fig 2D) and what exactly the dots in the scatterplots represent. This is necessary information to judge the quality of the presented results.

We clarified this question in the data analysis section in methods (lines 213-214):

“We required a minimum of eight consecutive bursts that could be detected for a sample recording to be accepted into the analysis.”

- Terminology: in a vast majority of publications on membrane conductances and bursting activities the persistent sodium current is referred as INap. Here it is called Ip. It would be judicious to use the same terminology as commonly used.

We agreed with this suggestion and changed IP to INaP throughout the text and Figures.

In the same vain, the authors named their two main bursting regimes as LVA and HVA for Low Voltage Amplitude and High Voltage Amplitude, respectively. Here again those acronyms are currently used for low and high voltage activated calcium current. To avoid any confusion would it be possible for the authors to propose another name.

We agreed and changed LVA and HVA to LA and VA throughout the text and Figures.

- page 16: The authors are able to reinstate bursting activity in HN neurons when recorded with a sharp electrode, an approach known to prevent bursting activity in these neurons and to induce a tonic discharge instead. They do this by introducing relatively small INap and Ipump current with dynamic clamp. What is the relevance of doing so? What are the interpretation of the authors to explain this effect? What is the functional link between INap+Ipump vs the shunting leak current supposedly activated by the recording sharp micropipette?

The experimental conditions are such that bursting is suppressed by the procedure.

We investigated a new mechanism supporting bursting activity suggested by the biophysical model; and found that now this interaction produces the bursting activity which is very similar to usual bursting activity assessed with the extracellular electrodes. At these low values of Ipump, the underlying mechanism compensates the effect of the depolarizing shunting leak current (with the reversal potential 0 mV) introduced by microelectrode. The small INap and Ipump current introduced by the dynamic clamp counteracts the depolarizing effects of the shunting leak current introduced by the recording sharp micropipette, which allows the bursting activity to be reinstated. This finding suggests that this interaction creates a mechanism which flexibly supports bursting activity across a wide range of dynamical parameters. You can find our paragraph in the discussion (lines 623-626), which we updated with a sentence in lines 661-663:

“This finding suggests that this interaction creates a mechanism, which flexibly support bursting activity across a wide range of dynamical parameters.”

-Fig 1: the authors described the emergence of a new bursting regime with a higher spike frequency and a higher amplitude of the envelope underlying the oscillations. Does this specific discharge pattern exist in “real” HN interneurons? Does it correspond to any physiological activity? Can this pattern be expressed under a specific neuromodulatory environment? What would be the physiological role of this HVA bursting activity? The authors must at least discuss these points and should make an effort to associate this bursting activity with any functional role in the animal.

This new HA bursting might be invoked in the cases of high neuromodulation where some Na+-carrying currents are activated and pump current is reduced or is challenged with Na+ influx. This regime has not been demonstrated yet in experiments with the leech heartbeat HN neurons, and further investigation is needed to explore the full range of conditions that can lead to the generation of HA bursting activity in these neurons. This remark is included now in lines 703-708:

“The predicted new HA bursting might be invoked in the cases of high neuromodulation where the Na+-carrying currents are activated, and pump current is reduced or is challenged with Na+ influx. This regime has not been demonstrated yet in experiments with the leech heartbeat HN neurons, and further investigation is needed to explore the full range of conditions that can lead to the generation of HA bursting activity in these neurons.”

- page 26: the authors propose that in bursting neurons the pump can potentially contribute to cessation of the burst phase and prolongation of the interburst interval and must be considered as part of the endogenous bursting mechanism. Then how does this interfere with the other conductances involved in burst termination (such as the different potassium associated conductances)? How can the authors be sure that during HVA and LVA bursting activities completely distinct sets of conductances can produce either high amplitude high frequency bursts vs low amplitude low frequency ones, especially when membrane conductances involved are voltage-dependent?

Thank you for this very important question. It is in the focus of our attention and we have further studies in preparation for further clarification as you can find in the lines starting at 752.

“We propose that there are at least two different bursting patterns, and that the roles of ionic currents changes with the switch between LA and HA potentially through neuromodulation. We dissected this new HA bursting mechanism down to interaction INaP and Ipump through [Na+]i, which allowed us to quantitatively match temporal properties like burst duration and interburst interval along with the change of the maximal pump activity. Since this description quantitatively describes changes of the burst duration, we conclude that this interaction controls the termination of the burst and interburst interval. Since, this mechanism produces shorter bursts and interburst intervals, it overrides the mechanisms described for LA bursting (Hill et al., 2001).

Minor:

- Please include line numbers

Done

- p5: please add the amplifier’s switching frequency in DCC.

Please, find this information with the abbreviation DCC included in line 119:

All intracellular traces were acquired at a sampling rate greater than 3 kHz using an Axoclamp 2A amplifier (Molecular Devices; www.moleculardevices.com) in discontinuous current-clamp (DCC) mode.

- p6: I am a little lost here, please clarify how the native currents are calculated. Are they estimated based on Hodgkin-Huxley equations and if so, which parameters were used? I don’t see any parameters or equations for the native currents in the dynamic clamp model in the supplementary data (p51ff), except I_NaF. And please state explicitly that the native currents are just estimates if that is the case.

We corrected the sentence in line 143:

The dynamic clamp also estimates the cell’s native fast Na+ current (spike-generating current) (INaF), native persistent Na+ current, (INaP native) and native pump current, (Ipump native) along with the injected INaP and Ipump to then estimate the intracellular Na+ concentration ([Na+]i) using Equation 1.

Thank you for catching this error, we added the INaP term to the equations describing the dynamic clamp model describing the full model.

- p7: Please add the duration of the dynamic clamp. How was steady-state determined after starting dynamic clamp or changing dynamic clamp parameters?

We added following passage in lines 121-124:

“After impaling the neuron with the sharp microelectrode, we injected a steady negative current (-0.3 nA) into the neuron for approximately 3 minutes, then progressively removed the injected bias current in steps of 0.01 nA until the magnitude of the injected current was reduced to -0.1 nA; the neuron was kept with -0.1 nA of steady current for the remainder of the experiment. Subsequently, if the neuron had an endogenous rhythmic bursting, the dynamic clamp protocol was initiated.”

- page 8, first para, last sentence: “The” is repeated twice.

Thank you, done.

- p8: “a two-dimensional model”. Please state here what the two dimensions are. It becomes clear later in the manuscript but please add it here.

We incorporated the information on the state variables of the two-dimensional model in the lines starting at 221.

“a two-dimensional model describing interaction of the membrane potential and intracellular Na+ concentration...”

- p8: “we gathered the following characteristics”. Cycle period is missing in this list.

We incorporated “cycle period (CP=BD+IBI),” into the line 230 as requested.

- p8: same paragraph: Please clarify how the data was normalized: Within each individual, or was each individual normalized to the mean of the population, etc.

Please, find the following in lines 236:

“Normalization was performed within each individual.”

- p8: Machine learning algorithms: Please provide the software.

We revised the manuscript by providing information regarding software and editing for clarity of the method description within a paragraph starting with line 240.

“An expert assisted in discriminating the two regimes with the two-threshold algorithm on 35% of the randomly selected data points by choosing the thresholds. As the result, the classification algorithm assigned a value of ‘1’ to the neuron sample activity if both the membrane potential amplitude and intracellular sodium concentration amplitude were larger than the respective thresholds of 20 mV and 1 mM, indicating high amplitude bursting activity. Conversely, a value of ‘0’ was assigned if either or both of the amplitudes were below or equal to their respective thresholds, indicating low amplitude bursting activity. The output of the two-threshold algorithm was used to train a Gaussian Naïve-Bayes supervised machine learning (ML) algorithm, which then was applied to classify the remaining 65% of the data. The ML algorithm was implemented in Python 3.6.8 with the Scikit-learn 1.1.3 library obtained from the Anaconda distribution (anaconda.com); and further details about the Naive Bayes algorithm can be found in the scikit-learn documentation (https://scikit-learn.org/stable/modules/naive_bayes.html).”

- p8: How did the authors evaluate the ML algorithm’s performance? Figure 2B clearly shows that the experimental design is unbalanced which can potentially lead to overclassification in a certain direction. If they did not evaluate the performance this point should go in the Discussion.

Thank you for your question. We appreciate your careful review of our work. To clarify, our experimental design was actually balanced across the two parameters of the dynamic clamp, as shown in Figure 2D. However, we understand how the reviewer may have interpreted Figure 2B as indicating an unbalanced design.

Regarding the evaluation of the ML algorithm’s performance, we agree that the potential for overclassification is an important consideration. We used standard metrics such as accuracy, precision, recall, and F1 score to evaluate the classification results as described above in response to the concern referring to p. 16.

- p9: linear regression: Please provide the software.

We provided this information in lines starting at 257 :

“All statistical analyses, including linear regression and non-parametric Mann Whitney U test were performed using Python 3.6.8 and Scipy 1.8 stats from the Anaconda distribution (anaconda.com).”

- p10: Equation 3: What is the division between Na and K components of leak?

As you can see in the moved Table 4 the conductances are g ®_(leak-K) = 8.8 nS g ®_(leak-Na)= 1.1 nS and the reversal potentials are -0.07 V for potassium (EK) and dynamic for sodium, computed as the sodium Nernst potential (ENa) depending on intracellular sodium concentration.

- p10ff: Please swap tables 2 and 3 so that they appear in the correct order in the text.

Thank you for the recommendation, we swapped the tables, we also moved Supplementary table 1 into the main text as table 1.

- p12: Why was -45 mV chosen as threshold?

We followed the definition of the depolarized and hyperpolarized phases established in our previous publication for experimental and modeling traces as reflected in our revised sentence in lines 303-307:

To divide the cycle period into the phases corresponding to the bursting waveform, we set a threshold at -45 mV and considered the depolarized and hyperpolarized phases as corresponding to the burst and silent phases, respectively following the definition of the phases established in Ellingson et al. 2021 (Ellingson et al., 2021).

- p14: I am confused by the n as to how many animals were used for which protocol. In the methods section, the authors state that data was normalized to Ipump_max = 0.3nA, g_P = 6nS. Here, there is only an n = 3 for the constant Ipump_max = 0.3nA and an n = 18 for g_P = 6nS. So how many animals were there in total, were they used for either constant I_pump or constant g_P or were both done in each animal?

Thank you for this important request. To clarify your question, some animals were used for both protocols, constant g ®_P or constant I_max^pump, and some others only for one protocol. These differences were not voluntary, but rather determined by how long the recorded interneuron was able to function after penetration by the sharp microelectrode. We clarified this issue by the following text in the lines starting at 347:

“We introduced the two currents with the dynamic clamp and used two different protocols to investigate the effect of varying g ®_NaP and I_max^pump on activity of the interneuron. For the first protocol, we kept I_max^pump constant at a value taken from the range [0.1 nA 0.9 nA] (N=40) and randomly varied g ®_NaP within the range between 1.0 nS and 7.0 nS. For the second protocol, we kept g ®_NaP constant at 6 nS (N=13) and varied I_max^pump within the range between 0.1 nA and 0.9 nA in random order. Some animals were used for both protocols if the recorded interneuron remained functional for a sufficient length of time. In total, 41 animals were used for this study.”

- page 15: rewrite the sentence: “The code can will be......”

This sentence is not in the manuscript and will be fixed in the process of submission.

- p15: “spike frequency similar to that obtained in extracellular recordings”. This statement does not match the data from the presented experiments and the literature on p16 where LVA spike frequency = 4.1 Hz, HVA spike frequency = 17.11 Hz and Cymbalyuk et al. (2002) spike frequency = 11.4 Hz. At least for spike frq it looks like those given in the literature are right between LVA and HVA.

Thank you for noticing this mismatch. We corrected the statement starting at line 361:” The reestablished bursting activity has properties typical for HN bursting: low voltage envelope amplitude underlying the slow membrane potential oscillations and the spike frequency similar to that obtained in intracellular recordings from neurons incorporated in a half center oscillator (Figure 1A) (Tobin and Calabrese, 2005; Kueh et al., 2016).”

- p15: “dynamic clamp currents [...] support rhythmic bursting [...] undeterred by the nonspecific leak”. This statement cannot be made at this point in the manuscript since the data about the impact of I_P and I_pump is presented later. So far, the authors have only shown that I_P and I_pump injection counteract the shunting leak.

For clarity, we removed this statement in this place.

- p15: “at a constant value (Figure 1 A-C)”. Contrary to the text, panel C has a higher Ipump_max than panels A and B.

We corrected this reference to the figure (lines 370):” Using the first dynamic clamp protocol, we systematically increased g ®_NaP while keeping I_max^pump at a constant value (Figure 1 A, B, D2).”

-page 17, line 13: are you sure that the reference to Figure 1B is appropriate? Shouldn’t it rather be Fig1A and C?

The reference to Figure 1B is correct. We made a more specific reference to HA bursting in figure 1D2.

- p17: “to terminate bursts”. The authors have not shown at this point in the manuscript that the pump can terminate the bursts, only that there is a correlation between I_pump levels and burst termination, as they say in the same sentence. Please rephrase. The evidence comes later, when they manipulated I_pump and analyzed the model phase plane.

We rephrased these sentences (lines 449-452):

“Due to its [Na+]i-dependence, the Na+/K+ pump responds to an increase in intracellular sodium concentration ([Na+]i) and could terminate bursts. Demonstrating that the peaks of the oscillations of Ipump coincide with the last spike of the burst, figure 3 agrees with this mechanism of burst termination.”

- p18: “kept all parameters constant”. Add “other” before parameters.

Done

- p18: How can there be cell-to-cell variability if all parameters are constant?

A simplified model could represent different experimental recordings with appropriate choice of the free parameters to represent cell-to-cell and preparation to preparation variability.

- p24: “taken care by”. Add “of”.

Done

- p27: “Because neurons communicate through spikes”. I have two comments about this section 1) Please differentiate here. Neurons also communicate through graded potentials (e.g. see Rosenbaum & Marder 2018 (https://doi.org/10.1523/JNEUROSCI.2632-17.2018) for a recent example), so the changes in the underlying voltage waveform could already be sufficient to support different functions. 2) Was HVA bursting ever observed under physiological conditions or is this some highly artificial state in which the neuron is driven by dynamic clamp?

In response to query #1, we were thinking of systems like the leech heartbeat CPG where HN neurons project for many segments/ganglia engaging both other interneurons of the CPG and motor neurons through inhibitory synapses and electrical junctions. Segmental interactions are of limited importance consisting of half-centers in only 2 segments. We have revised accordingly starting with line 679-681.

“Because HN neurons project several segments/ganglia to engage other HN neurons of the CPG and motor neurons through inhibitory synapses and electrical junctions, spike are the major mode of communication in the network, thus the differences...”

In response to query #2, please see our response to Major Concern regarding Fig 1. Above repeated here for clarity (added starting line 703).

“The predicted new HA bursting might be invoked in the cases of high neuromodulation where the Na+-carrying currents are activated, and pump current is reduced or is challenged with Na+ influx. This regime has not been demonstrated yet in experiments with the leech heartbeat HN neurons, and further investigation is needed to explore the full range of conditions that can lead to the generation of HA bursting activity in these neurons.”

- p51, 52: What is denoted by f_infinity in the equations of m and h gates? I assume it is a logistic function but what does it look like exactly and which of the input parameters in the parentheses is the half-activation voltage and which is steepness?

We added definition of this function in lines 164-166:

“Dynamic clamp model is described by the following system of ordinary differential equations with the parameters provided in the table 1 with the steady state activation and inactivation functions described by the Boltzmann function f_∞ (A,B,V)=1/(1+e^(A⋅(V-B))) .”

- Figure 1:

o Avoid unnecessary color coding, e.g. the bars for T, BD, IBI, the mid-burst diamonds and the spike frq traces should all be black.

Color avoided, as suggested.

- Remove the CV in panel B because this can lead to some confusion with the CVs for cycle period that are in the figure legend. Make it consistent with panels A and C and simply state that this is irregular LVA.

CV deleted, HA and LA labels applied, and Irregular HA label applied.

- The CVs for cycle period are nowhere mentioned in the text. Why are they there, what are they supposed to tell me?

CV deleted; see text.

-add LVA and HVA above traces in panels D1 and D2, respectively.

HA and LA labels applied.

- Figure 2:

- What do the individual dots in panels B and C represent? Are these the averages for each animal of x cycles, or just for one cycle?

The averages for each animal of x cycles

Does each dot come from one combination of I_P and I_pump?

Yes.

- Figure 3:

- Avoid unnecessary color coding and using the same colors for different things: In the previous figures, red was for HVA and blue for LVA. Here, it is for Vm and I_P. The color does not add any information. Either make all traces black or color the Vm trace according to LVA or HVA.

Color adjusted just Voltage trace is colored to reflect HA (red).

- Figure 4:

- Colors are completely unnecessary here since every individual shows the same trend and the colors are so similar that it is difficult to follow a single experiment. Show only the mean line with shaded confidence intervals.

We respectfully disagree. The trends are the same, but we wish to show the variability across preparations. Colors and individual lines retained.

- Swap panels E and F so they appear in the same order as in the text.

Done.

- Figure 5:

- If you use colors, use a consistent color code. Previously, cyan was used for the voltage envelope of HVA bursting. Here, it is yellow; and cyan is used for the model data.

Color code adjusted to be consistent. Panels A and B in black.

- Supplementary Figures 1 and 2

- Please use colors that are different from

Colors adjusted.
